# A Six-Membered Concerted Mechanism for CO_2_ Capture by Amines Studied under Charged Microdroplet Reaction Conditions

**DOI:** 10.1021/acs.analchem.6c00175

**Published:** 2026-04-22

**Authors:** Taghi Sahraeian, Dmytro S. Kulyk, Ayesha Seth, Glib V. Baryshnikov, Abraham K. Badu-Tawiah

**Affiliations:** † Department of Chemistry and Biochemistry, 2647 The Ohio State University, 100 West 18th Ave, Columbus, Ohio 43210, United States; ‡ Laboratory of Organic Electronics, Department of Science and Technology, 4566 Linköping University, Norrköping SE-60174, Sweden

## Abstract

Carbon dioxide (CO_2_) capture and storage represents an important technological challenge. A mechanistic understanding of interactions involved in the capture process is necessary not only for technological development but also for efficient conversion of captured CO_2_ into value-added materials. Herein, we present a novel contained secondary electrospray ionization platform for studying the interactions of gaseous amines and CO_2_ gas under microdroplet reaction conditions, which enables mass spectrometry (MS) characterization of CO_2_ capture products and intermediates in real time. We detected [2 M + CO_2_ + H]^+^ species, which corresponds to a six-membered intermediate. DFT calculations confirmed the high stability of the protonated six-membered ring intermediate. This finding provides a plausible concerted mechanism in the microdroplet environment that excludes the involvement of thermodynamically disfavored ionic species. The carbamic acid counterpart of the final product/salt was readily characterized by tandem MS. The carbamic acid/amine salt was also isolated and characterized by Fourier transform infrared spectroscopy. By virtue of the fact that headspace vapors of amines are sampled, we were able to establish a high-throughput platform that enabled the CO_2_ capture capacity of five different amines to be studied in under 2 min. The same device also enabled the absolute quantification of capture capacity.

## Graphical abstract

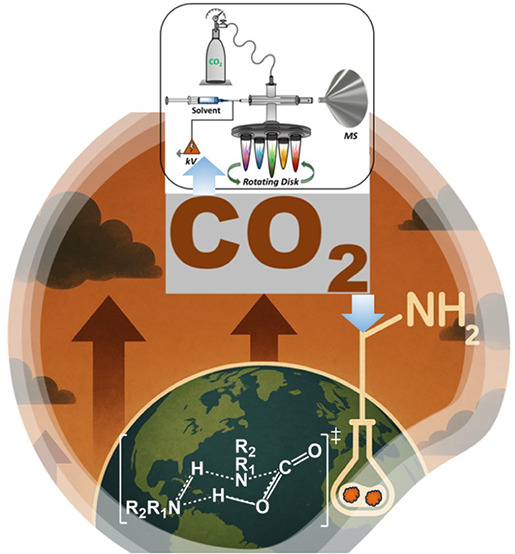

## Introduction

With global fossil CO_2_ emissions now exceeding 37 billion metric tons annually,^1^ the urgent need for efficient mitigation and valorization strategies is undeniable; among them, amine-based approaches are particularly important because of their high reactivity and selectivity with CO_2_, tunable chemistry, and established industrial relevance.^2^ Although the ability of amines to capture CO_2_ is well-known, the mechanism describing the initial interaction between the two species is still being debated. In a series of studies conducted under different experimental conditions (temperature, pressure, different solvents, and amine types), Kortunov and coworkers used nuclear magnetic resonance to systematically investigate the underlying chemistry between the amine and CO_2_.^3–5^ These studies led to the suggestion that the first step involves a nucleophilic attack on CO_2_ by the amine to form a zwitterion, RH_2_N^+^–COO^–^. The zwitterion was proposed to be the intermediate for a four-membered mechanism (1,3-zwitterion; Scheme 1A) through a concerted reaction with synchronous formation of C–N and O–H bonds. To date, the zwitterion intermediate involved in this concerted reaction has not been detected.

**Scheme 1:**
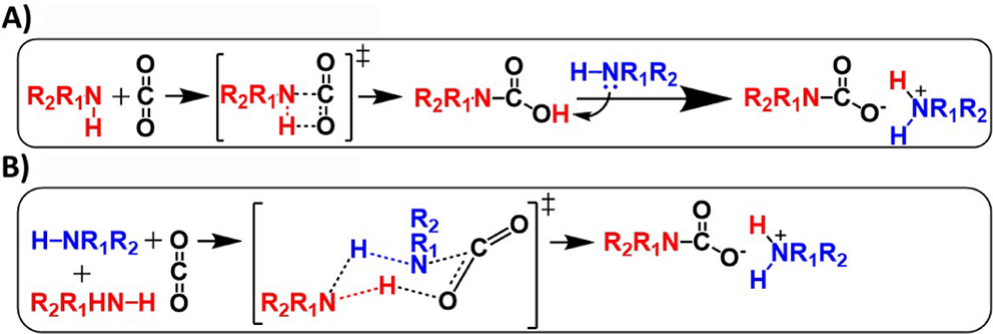
CO_2_ Capture Mechanism by Amines through (A) Four-Membered and (B) Six-Membered Intermediates

Concurrent theoretical studies by multiple authors^6–9^ suggested that the energy barrier required for the formation of carbamic acid using two molecules of amines *per* one CO_2_ molecule was substantially lower (16 kcal/mol) than when one amine molecule *per* one CO_2_ was used (energies ranged 40–50 kcal/mol). The Gibb’s free energy of activation for the four-membered intermediate was calculated to be 42.8 kcal/mol,^10^ which is within the energy range for reactions involving 1:1 amine and CO_2_. Results suggesting lower activation energy for CO_2_ capture processes involving two molecules of amine have been reproduced in systems that used one amine and an assisting species such as water.^10^ Such amine/CO_2_ reactions assisted by another amine (or water) are proposed to proceed through a six-membered intermediate (1,5-zwitterion), as described in Scheme 1B. Amine-functionalized surfaces such as silica and metal–organic framework are proposed to capture CO_2_ *via* a bridging mechanism that involves two amine groups.^11,12^ For solution-phase and gas-phase systems, evidence for the existence of such a six-membered mechanism is lacking. Isotope experiments^10^ characterized by Fourier transform infrared (**FTIR**) spectroscopy showed indirect evidence for this six-membered mechanism, but direct capture of such species has not yet been reported. Such data will offer important molecular information and provide definitive insight into the mechanism involved. Therefore, the development of a soft, rapid, and *online* analytical method that can directly detect and differentiate 1,3-zwitterion (four-membered) and 1,5-zwitterion (six-membered) intermediates would be most useful. Herein, we show how the combination of computational data with a new mass spectrometry method allows the differentiation of three closely related species, all related to the six-membered intermediate.

Mass spectrometry (MS) has the capacity to provide both qualitative and quantitative information about chemical substances. Although MS could be used to analyze products and byproducts from *off-line* absorbent columns, an *online* reaction screening coupled with *in situ* MS will not only improve mechanistic understanding but also expedite the discovery of new absorbent materials. For example, while monoethanolamine (MEA) scrubbing is considered one of the most efficient and economical approaches for postcombustion CO_2_ capture, this method has some major drawbacks, including low stoichiometric capture capacity (0.5 mol CO_2_ *per* mol of MEA), high energy requirements for regeneration, corrosiveness, volatility, and instability. Such limitations encourage further studies in the search for other amines that can mitigate some of these challenges. No such technique exists that can offer mechanistic insights *via* instantaneous detection of the CO_2_-amine products and intermediates, as well as providing opportunities to evaluate the capture capacity of different amines (and other absorbents) in a high-throughput fashion.

In the present work, we adapted and validated a secondary electrospray ionization (SESI)^13,14^ MS setup, as a reactor/ion source for the *online* screening of amine capture capacity and for studying the CO_2_ capture mechanism. We introduced novel “containment” systems to the SESI experiment to enable the effective introduction of amine as a headspace vapor and CO_2_ as an electrospray nebulizer gas. Therefore, we refer to the reaction platform as contained-SESI, which is an important continuation of our efforts to create new contained (multifunctional) ion sources.^15–23^ In addition to the ability to introduce multiple gases, the contained-SESI platform also included a reaction cavity at the outlet of the electrospray emitter to enhance capture, mixing, and reaction of the two vapors under the charged microdroplet environment. These features make our experiment reactive, which we also term reactive contained-SESI.

Reaction acceleration in charged microdroplet has recently gained popularity because of its importance in chemical synthesis, reaction monitoring, and elucidation of mechanisms *via* the capture of transient reaction intermediates.^24–36^ Enhanced surface effects and the concentration of reactants in the tiny microdroplets are mainly responsible for the observed rate enhancements. Recently, Cooks’ research group utilized electrosonic spray ionization (ESSI) to promote CO_2_ reactions with amines present at the surface of microdroplets to form carbamic acid products in a gas/liquid reaction system.^37^ The mechanism of carbamic acid formation was ascribed to superacid or superbase catalysis at the air/water (microdroplet) interface. A related report^38^ used bicarbonates added to electrospray solvent to generate CO_2_ microbubbles within the charged microdroplets. This was found to facilitate amine/CO_2_ reactions for subsequent *online* MS characterization of the carbamic acid products. The internal microbubble formation increased the total area of the liquid–gas interface, which resulted in higher conversion ratios compared with experiments based on ESSI. Surprisingly, the bicarbonate-based microbubble could not accelerate the reactions of primary amines with CO_2_. Both experiments (microbubble and ESSI) utilized millimolar (mM) concentrations of amines, a quantity that may be prohibitively large for preliminary reaction screening in search of new absorbents. Recent research has expanded CO_2_ capture in charged microdroplets beyond amines, for instance, shifting focus toward nitrogen heterocycles^39^ or the exotic iodopentafluorobenzene radical anion.^40^ As stated earlier, our contained-SESI platform utilizes headspace vapor (∼20 μM) of amines for reaction with CO_2_. The ability to use minuscule vapors of amines enables our contained-SESI platform to be operated in a high-throughput fashion, without significant carryover.

The traditional SESI^13,14^ experiment is based on the ionization of neutral gaseous molecules by charged microdroplets derived from electrospray ionization (**ESI**). SESI is a softer spray-based ion source compared to ESI since it generates ions with a lower average internal energy.^41^ However, the challenge of capturing analyte vapor with charged microdroplets for on-the-fly ionization makes SESI less efficient as an ion source compared to ESI, where the analyte is added to the spray solution. In principle, microdroplet-based reaction acceleration can be expected under SESI experimental conditions. Aside from charged microdroplets, liquid thin films can also increase the reaction rate through reagent concentration *via* solvent evaporation and therefore have been exploited for this purpose.^42–44^ The reaction cavity included at the outlet of the contained SESI platform enables reactions to be accelerated in both microdroplets and thin liquid films in a single experiment. All reagents were delivered as vapors and not added to the spray solvent. This makes our experiment different from other recent SESI studies, where the modifying reagent (Girard T) is added to the spray solvent (MeOH) to derivatize gas-phase aldehydes.^45^

The key concept in our work is related to gas-droplet interactions within a discontinuous thin film, as opposed to reactions in the pure droplet phase. The dissolution of two gaseous molecules into a thin liquid film created in contained-SESI and its subsequent transformation—within milliseconds time scale—into a spray of secondary droplets affords an avenue for both liquid- and gas-phase reactions. As a derivative of the conventional SESI, our contained-SESI process is softer than ESI, which makes it a more suitable process to study reversible reactions and to detect labile, noncovalently bound intermediates. We show data for CO_2_ capture by different amines, which displayed different capture capacities based on molecular size (smaller compounds were favored). For the first time, we provide specific molecular data that suggest the involvement of the six-membered intermediate. Our experiments proved that CO_2_ is not simply absorbed onto the amine molecule during the capture process based on charged droplets. Finally, we demonstrated high-throughput screening of amines *via* the use of a rotating disc, which allowed headspace vapors of different amines, with varying vapor pressures, to be sampled and reacted with atmospheric CO_2_.

## Experimental Section

### Mass Spectrometry

The mass spectrometer used was a Thermo Fisher Scientific Velos Pro LTQ mass spectrometer (San Jose, CA, USA). MS parameters employed, unless otherwise stated, were as follows: 250 °C inlet capillary temperature, 5 kV spray voltage, 3 microscans, 100 ms ion injection time, and 60% S-lens voltage. Spectra were obtained for at least 30 s, providing an average of 300 individual scans. MS data collection and processing were performed using Thermo Fisher Scientific Xcalibur 2.2 SP1 software. Tandem MS with collision-induced dissociation (CID) was executed for analyte identification. For CID, the isolation window of 1.5 Th (mass/charge units) and a normalized collision energy of 30% (manufacturer’s unit) were chosen.

### Solid-State Fourier Transform Infrared (FTIR) Spectroscopy

FTIR spectra of the white solid residue resulting from the exposure of benzylamine to CO_2_ gas using our reactive contained-SESI platform were interrogated with a PerkinElmer Spectrum 3 infrared spectrometer with attenuated total reflectance accessory, which is a universal ATR Diamond-KRS-5 single-bounce assembly. Triplicate measurements were made with the same sample left on the crystal, and data were recorded one after another using MIR-TGS detector. A total of 32 spectral scans were conducted and averaged for each measurement between the 4000–380 cm^–1^ range with 4 cm^–1^ resolution.

### Chemicals, Reagents, and Samples

Propylamine (99%, extra pure) and 3-phenylpropylamine (≥98%) were purchased from Fisher Scientific (USA). Butylamine (99.5%), benzylamine (99%), hexylamine (99%), N,N-dibutyl-1,3-propanediamine (98%), methanol (99.9%, HPLC grade), acetonitrile (99.8%), and acetone (99.9%) were all obtained from Sigma-Aldrich (St. Louis, MO). Pure N_2_ and ultrapure CO_2_ were used for the corresponding experiments. All aqueous solutions were prepared in 18.2 MΩ water from a Milli-Q water purification system (Millipore, Billerica, MA). Borosilicate capillaries (ID 1.17 mm) were provided by Sutter Industries (Novato, CA, USA).

## Results and Discussion

### Contained-SESI MS

The setup for contained-SESI is as illustrated in Figure 1. The apparatus consolidates two operating systems into a single platform. The first component is the ESI emitter that generates charged microdroplets from a suitable spray solvent (e.g., methanol). The second component involves the containment features, which allow (1) the headspace vapor of the amine to be sampled by the rapidly moving (∼100 m/s)^46^ charged microdroplets and (2) the inclusion of a cavity with adjustable length at the outlet of the ESI emitter. Instead of the typical N_2_, we utilized CO_2_ in the contained-SESI experiment, which served both as the nebulizer gas and as a reactant with the amine vapor. The direction of flow of the CO_2_ gas within the cross Swagelok element facilitates the release of the amine vapor (from the liquid amine), which is subsequently swept into the electrosprayed charged microdroplets inside the reaction cavity. This process sets contained-SESI apart from other methods in that it provides two coexisting but unique conditions for CO_2_ capture/reaction: (a) droplet-phase reaction, where the two gas-phase species interact, and (b) liquid-phase, where dissolved reactants interact in a liquid thin film. Both the microdroplet and thin film reaction systems are capable of reaction acceleration. To the best of authors’ knowledge, the concomitant use of droplet and liquid thin films for CO_2_ capture has not been reported before. A third unique feature of our contained-SESI setup is its ability to enable a fraction of the CO_2_ gas to be dissolved into the original liquid amine (i.e., starting amine reagent) during the initial encounter in the container. The dissolution of the CO_2_ in the amine results in a phase change from liquid to solid phase, allowing the reaction product to be characterized not only by MS alone but also by other analytical techniques such as infrared spectroscopy. The mass change of the initial liquid amine in this process directly yields the capture capacity for different amines.

**Figure 1:**
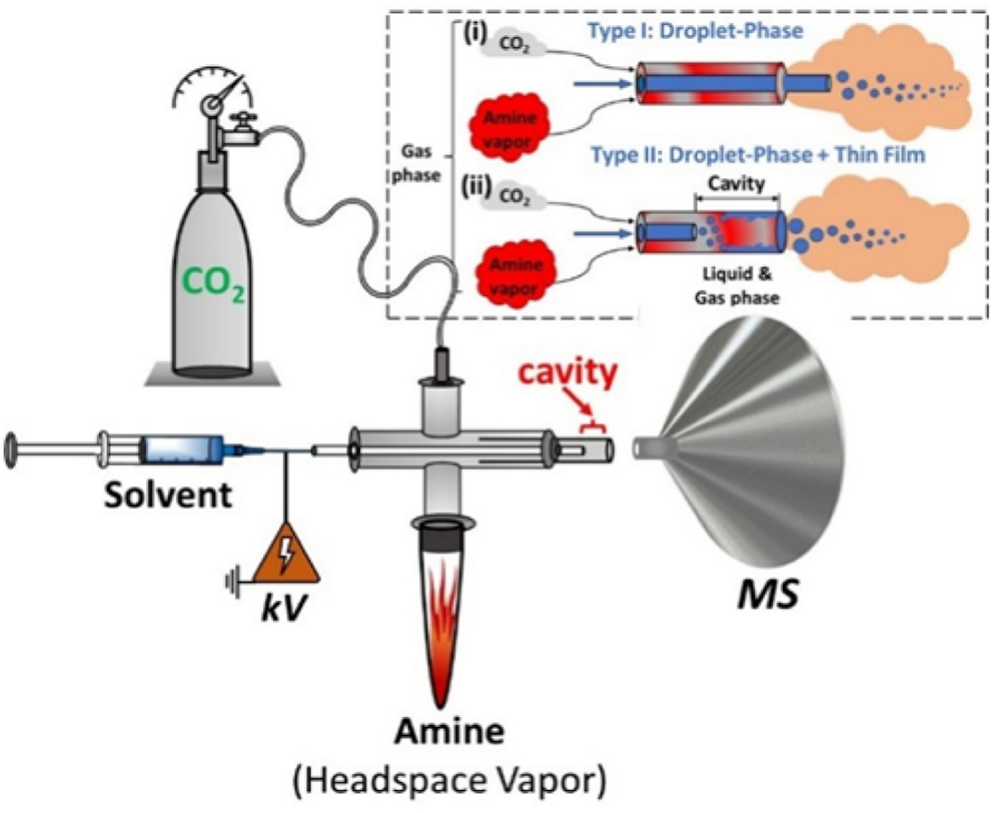
Experimental setup for contained-SESI MS for the capture of CO_2_ by primary amine vapor, followed by *online* product and intermediate detection by a proximal mass spectrometer. The platform was operated in two modes: Type I mode, with the inner capillary protruding from the outer capillary (inset i) and Type II mode, with the inner capillary regressed inside the outer capillary, creating a reaction cavity (inset ii).

The contained-SESI emitter was constructed from a cross Swagelok unit (Figure 1) that allows three inputs: delivery of the spray solvent through the fused silica (FS) capillary, CO_2_ reagent gas, and amine headspace vapor. The spray solvent consists of a blank MeOH or MeOH/H_2_O (1:1, v/v) solution. The ESI emitter itself comprises of a concentric pair of inner (100 μm ID FS) and outer (250 μm ID FS) capillaries. The inner FS capillary can adopt two configurations, which create two modes of operation: in Type I (insert (i), Figure 1), the capillary is pushed through the cross Swagelok element all the way to the outlet, where it is adjusted to be slightly (∼1 mm) outside of the outer capillary. In Type II (insert (ii) Figure 1), however, the inner FS capillary is regressed (≥1 mm) inside the outer capillary, forming a cavity of adjustable size based on how far the inner capillary is regressed inward.

As already noted, the cavity included at the outlet of the contained-ESSI source provides conditions where both liquid thin-film and droplet-phase reactions can occur. The outer FS capillary that makes it possible to create the cavity allows a pressure of <40 psi CO_2_ to be transmitted, which subsequently interacts with the spray solvent delivered from the inner FS capillary. Both the inner and outer FS capillaries are held in place by graphite ferrules. Electrosprayed charged microdroplets are created after applying a direct current (DC) high voltage (±5 kV) to the metal tip of the syringe connected to the inner FS capillary of the ESI emitter. The final microdroplets transfer the ionized amine vapor and reaction product resultant from CO_2_-amine interactions directly to the MS inlet for subsequent characterization.

Instead of using microdroplets to capture amine vapor, the amine can be dissolved into the spray solution. This experiment becomes a conventional ESI experiment, which is also available on our contained-SESI source. Therefore, four unique experiments are possible, which were fully explored in the current study: 1) Type I mode, where amine vapor is captured with charged microdroplets in contained-SESI in the absence of a reaction cavity, 2) Type II mode, in which amine vapor is captured and reacted with CO_2_ in contained-SESI in the presence of a reaction cavity, 3) Type III mode, where amine solution is analyzed (i.e., amine is added to the ESI spray solvent) without a reaction cavity, and 4) Type IV mode, in which amine solution is analyzed with a cavity included at the outlet of the source.

### Capture of Six-Membered Intermediate

Figure 2A shows a negative-ion mode mass spectrum recorded when the headspace vapor of benzylamine (MW 107 Da) was exposed to CO_2_ gas under the charged microdroplet environment. The CO_2_ capture product, benzylcarbamic acid, was detected as a deprotonated species at *m*/*z* 150. The identity of the species at *m*/*z* 150 was confirmed by tandem MS (MS/MS) (Figure 2B) by applying collision-induced dissociation (CID), in which it underwent two major competitive fragmentations. The first involved water loss from the parent ion to give a fragment at *m*/*z* 132, which subsequently dissociated to a fragment ion at *m*/*z* 104 *via* CO loss. The second competitive dissociation pathway involved the loss of CO_2_ from the parent ion to give a fragment ion at *m*/*z* 106. These unique fragmentation patterns confirm the expected structure of the benzylcarbamate anion. But most importantly, the MS/MS studies reveal the presence of a relatively strong covalent bond between CO_2_ and the amine; a simple adduct formation will result in the uncompetitive loss of CO_2_ from the corresponding anion. The observed fragmentation pattern has been reproduced for other carbamate products (Figure S1), including ethanolcarbamate (*m*/*z* 104), butylcarbamate (*m*/*z* 116), and 3-phenylpropylcarbamate (*m*/*z* 178).

**Figure 2:**
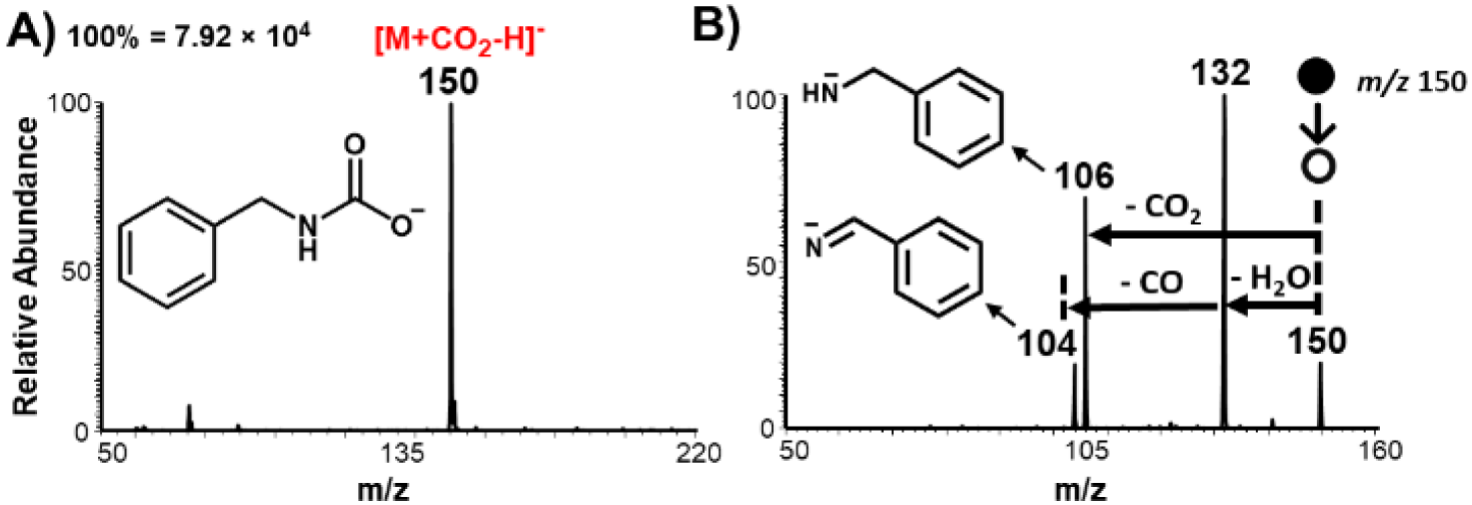
(A) Negative-ion mode contained-SESI MS spectrum (Type II mode) showing the presence of benzylcarbamic acid (*m*/*z* 150), which is derived from the interaction of headspace vapor from benzylamine with CO_2_ nebulizer gas. (B) Tandem MS of the product peak at *m*/*z* 150.

Interestingly, *online* MS evaluation in the positive-ion mode revealed the presence of the six-membered intermediate but not the four-membered species. These data are shown in Figure 3A, in which a protonated species is detected at *m*/*z* 259. Based on molecular weight, the chemical composition of this species is identified to include two molecules of benzylamine and one molecule of CO_2_. High-resolution Orbitrap analysis confirmed the elemental composition of the peak at *m*/*z* 259 to be [2 M + CO_2_ + H]^+^ species with <1.3 ppm accuracy (Figure S2A). The MS/MS analysis of this species (*m*/*z* 259) (Figure 3B) shows a rather complicated spectrum that will make sense when considering that the six-membered intermediate is formed *via* a network of three strong intermolecular forces that engage all three molecules involved (two amines and CO_2_). Again, a simple adduct would result in the elimination of individual molecules involved rather than the cross-ring breakages observed. For example, important pathways such as the loss of CO and H_2_O to give species at *m*/*z* 231 and 241, respectively, were observed. The diagnostic benzyl cation was also detected at *m*/*z* 91, which was accompanied by protonated benzylamine at *m*/*z* 108. Similar [2 M + CO_2_] species, which we assign to the presence of the six-membered intermediate, were detected for butylamine (Figure S3) and confirmed by high-resolution Orbitrap mass spectrometry, yielding a mass error of less than 1.7 ppm (Figure S2B). For benzylamine, the four-membered species [M + CO_2_] would be expected to show up at *m*/*z* 152 in the positive-ion mode, but this species is not detected for all amines tested. These results support previous suggestions that the presence of a second amine can significantly stabilize the intermediate involved, allowing its detection in the gas phase by a mass spectrometer as shown here. We noted that experimental parameters such as S-lens RF voltage and MS inlet capillary temperature influenced the appearance of positive-ion mode mass spectrum (Figures S4–S6), with lower values producing softer experimental conditions as also seen in other experiments.^28,41^

**Figure 3:**
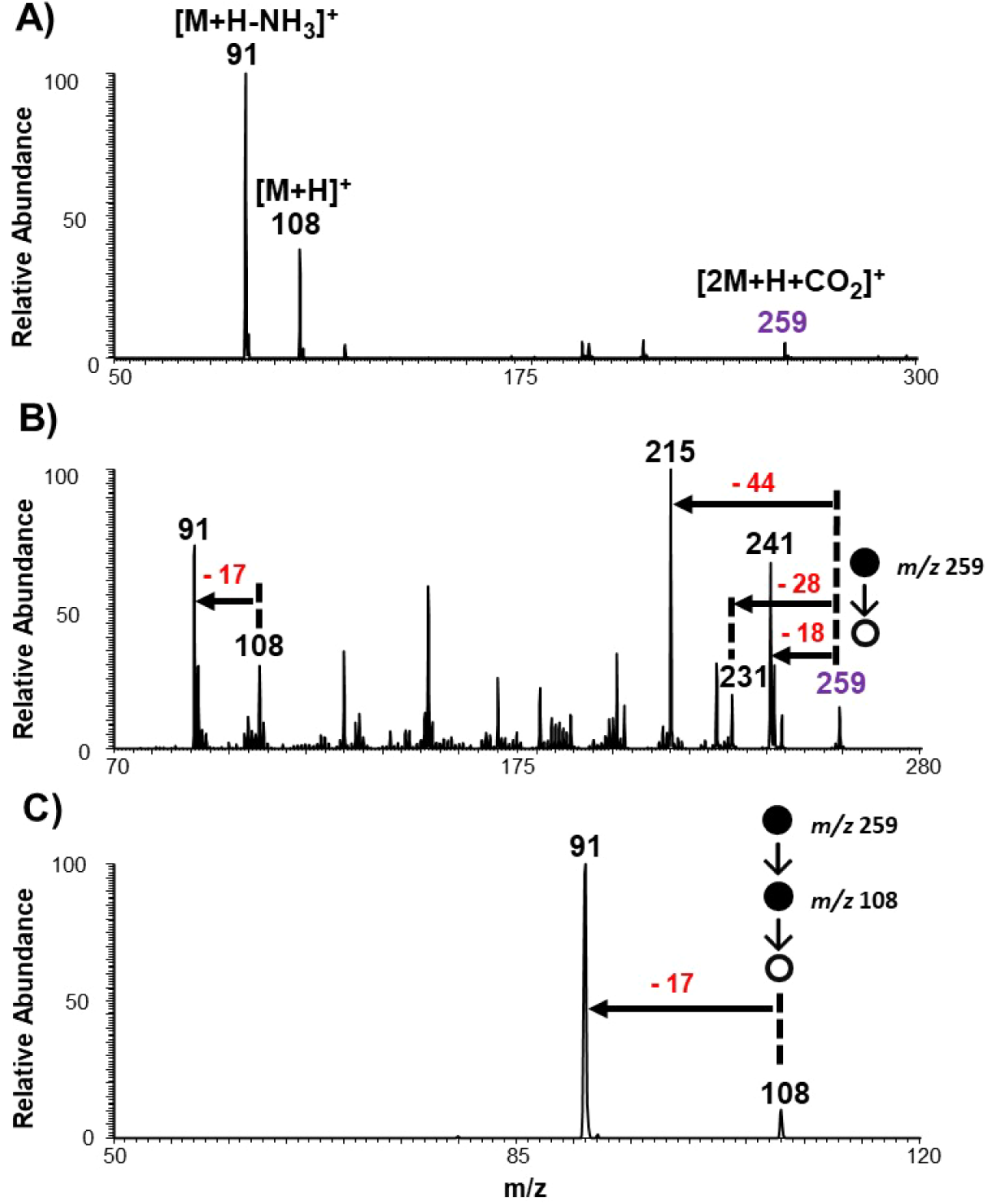
Identification of six-membered intermediate [2 M + CO_2_+H]^+^ from the reaction between benzylamine and ambient CO_2_. (A) Positive-ion mode mass spectrum of the benzylamine reaction with CO_2_. (B) Tandem (MS^2^) mass spectrum of the intermediate at *m*/*z* 259 and (C) the corresponding MS^3^ at *m*/*z* 259 and *m*/*z* 108.

### Solid Analysis by FTIR

The benzylcarbamic acid/benzylamine salt will form if CO_2_ is exposed to a liquid (neat) amine. This process is expected to occur inside the container attached to the contained SESI platform. This product has the same chemical composition as the proposed six-membered intermediate, which reversibly and spontaneously transforms into the respective amine and CO_2_ under ambient conditions, as observed by us over time. We isolated the solid (benzylcarbamic acid/benzylamine salt; Figure S7) and characterized it using solid-state FTIR. The resultant FTIR spectrophotogram shown in Figure 4B reveals that the product synthesized in our contained-SESI source following the exposure of benzylamine to CO_2_ gas has features/functional groups that are identical to the spectrum (Figure 4A) recorded from a standard benzylcarbamic acid/benzylamine salt found in the database created by the National Institute of Standards and Technology (NIST).^47^

**Figure 4:**
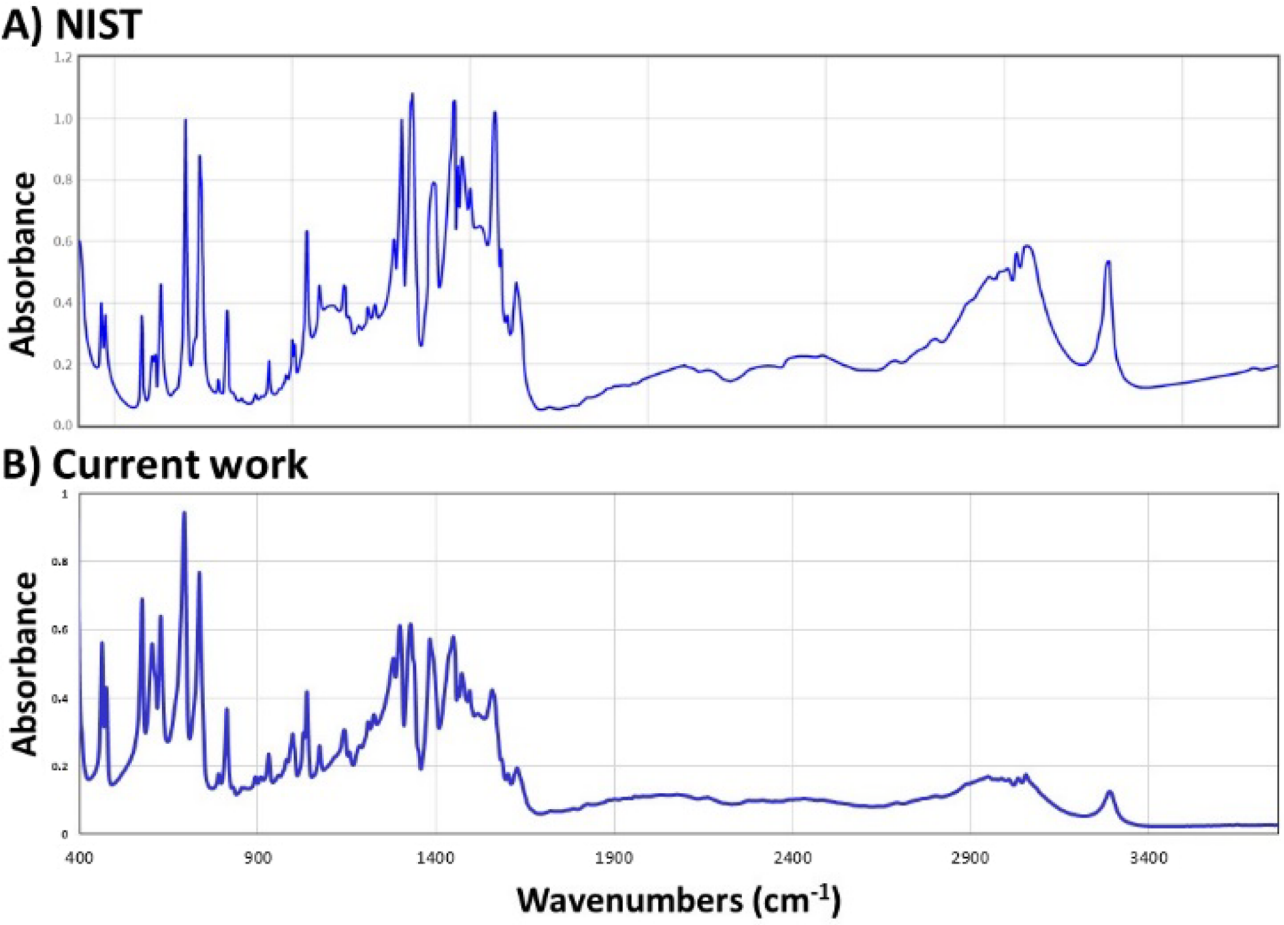
Solid-state Fourier transform infrared spectroscopy (FTIR) spectrophotogram for the white powder product benzylcarbamic acid/benzylamine salt derived from (A) a standard sample from NIST and (B) in-house product synthesis from the reaction of benzylamine liquid with CO_2_ gas.

For example, the presence of the amide functional group (−CO–NH– moiety) is revealed at around 814 and 464 cm^–1^ corresponding to amide V and amide VI bands, respectively (Figure S8). This confirms the conversion of the original benzylamine reactant (with −NH_2_ functional group) to benzylcarbamic acid containing the amide functional group after exposure to CO_2_. This experiment/data showcases the unique feature of our experiment: that is, because our contained-SESI platform enables gas/droplet interactions at atmospheric pressure, the ensuing CO_2_ capture products and intermediates can be analyzed on-the-fly by MS (Figure 3) *via* headspace analysis, or the solid products can be isolated and characterized as neat powder by other spectroscopic methods as demonstrated in Figure 4B.

Aside from direct analysis with FTIR, dissolution of the collected solid for subsequent analysis by MS is also possible, although the salt will dissociate to give the individual components, including dissolved CO_2_. MS analysis of the resultant solution showed a species at *m*/*z* 259 (Figure S9), which we assign to be the protonated six-membered complex [2 M + CO_2_]. The absence of applied voltage under sonic spray ionization conditions yielded a high intensity for the *m*/*z* 259 species. Although the mass of the six-membered intermediate is the same as the benzylcarbamic acid/benzylamine salt, the two species might exhibit markedly different stability in condensed phase *versus* gas phase. It is hard to conceive that the ionic interactions in benzylcarbamic acid/benzylamine salt can be maintained after being dissolved in methanol/water solution to enable intact transfer into the gas phase. Also, protonation will inevitably neutralize the carbamate component of the salt, which will subsequently weaken the interaction with the amine (discussed later by using DFT calculations). Therefore, we propose that the protonated [2 M + CO_2_] species detected from the analysis of the dissolved solid powder must be the six-membered intermediate that has originated from a reaction event occurring in the microdroplet environment between the dissolved amine and CO_2_. The CO_2_ used can be inherent to the original solution (after dissolving the solid) or can be captured from ambient air. The ability to capture atmospheric CO_2_ is discussed later for our high-throughput experiment. In any case, the fact that no indication of the four-membered intermediate [M + CO_2_] is observed for both *online* and *off-line* experiments supports suggestions that rule out this mechanism because of its high activation energy.

### Insight from Physico-Chemical Changes

The solidification of amine in the container, after exposure of the amine solution to CO_2_ gas, results in the release of heat. This exothermic process generates approximately 25 °C of heat. That is, the temperature of the container (originally at 25 °C) reached 50 °C. This temperature change is accompanied by physical and chemical changes, during which the colorless liquid benzylamine (Figure S10B) reagent turned into a white crystalline powder after about 3 min (Figure S10C). Reaction time was monitored visually through the transparent plastic material of the container. We also estimated the time it takes for the benzylamine in the container to fully form the solid product using selected ion monitoring (SIM) tandem MS of the protonated benzylamine (Figure S10A). Here, we chose to monitor the benzylamine reagent since it gets consumed during the reaction, and the end point (disappearance of this reagent) should be easier to determine. By considering the midpoint of the decay curve recorded for the protonated benzylamine signal, we determined the time required to fully react/solidify 1.0 mL of benzylamine to be 2.8 min, which matched the solidification time determined from visual observations. Collectively, the physical and chemical properties of the crystallized product as determined by solid-state FTIR, temperature, and color changes, all corroborate the identity and structure of the proposed benzylcarbamic acid/benzylamine salt ruling out potential adsorption processes, which might represent a mere physical trapping of CO_2_ by benzylamine. Direct analysis of the headspace vapor of the pure solid by the contained-SESI MS platform did not produce the [2 M + CO_2_] peak, indicating that direct desorption/ionization of the solid benzylcarbamic acid/benzylamine salt is inefficient.

### Other Important Mechanistic Considerations

Possible CO_2_ capture mechanisms by amines under the charged microdroplet experimental conditions were discussed recently in the literature^29,30^ and are depicted here in Scheme 2. Protic solvents are ionized during ESI to generate acids (H^+^) and bases (R^−^) that can either protonate CO_2_ in positive-ion mode or deprotonate an amine in negative-ion mode. Both of these events can facilitate nucleophilic attack on CO_2_ by amines to form either protonated (+ ion mode) or deprotonated (− ion mode) carbamic acid.^8^ We observed mostly deprotonated carbamic acids when using lower concentrations of amines (from headspace vapors), which suggests that the deprotonation mechanism could be favorable compared to the protonation mechanism when considering primary amines.

**Scheme 2:**
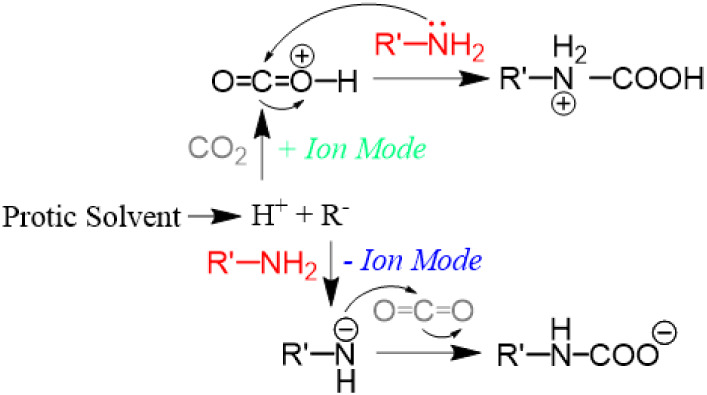
Previously Proposed Mechanisms for CO_2_ Capture by Amines in Charged Microdroplets in Positive- (+) and Negative (-) Ion Modes

This observation can be explained in two ways: First, deprotonated amine is a stronger nucleophile than neutral amine, and second, protonation in positive-ion mode will occur preferably for amine, not CO_2_ that has lower proton affinity (PA, 541 kJ/mol) compared to amines (i.e., PA of benzylamine is 913 kJ/mol). Obviously, the protonated amine cannot act as a nucleophile. The latter pathway in positive-ion mode also raises a logical thermodynamic question about how CO_2_ could be protonated by H_3_O^+^, since H_2_O species have a higher proton affinity (PA 725.6 kJ/mol) than CO_2_.

A third plausible mechanism is described in Scheme 1B, which occurs through the formation of a six-membered complex between the amine and CO_2_ without any assistance from acid/base catalysis. Although this mechanism requires some activation,^10^ its contribution to carbamate formation can be explained by the exothermic nature of the CO_2_-capture process. The formation of this six-membered intermediate provides a feasible reaction mechanism toward the formation of species detected as [2 M + CO_2_]. The concerted nature is consistent with accelerated reactions observed for electrosprayed droplets. Importantly, this mechanism can occur without regard to the polarity of the ionization process, which provides a more favorable alternative pathway for protonation of CO_2_ in positive-ion mode and deprotonation of amine in negative-ion mode. We performed a separate *off-line* experiment where thin films of benzylamine were exposed to 5 kV of positive and negative DC voltage and did not observe any difference in terms of reaction kinetics or thermodynamics compared to when no voltage was used. This suggests that the charged environment (including polarity) of the electrosprayed microdroplets plays a minor role. The fact that the crystalline salt forms readily in the container is additional evidence that the existence of charged intermediates, proposed in Scheme 2, might be less likely.

### DFT Studies

To confirm our experimental findings concerning the existence of a protonated six-membered intermediate, we performed computational geometry optimization (Table S1). This was followed by the analysis of electron density distribution for all collision complexes of benzylamine and CO_2_ using Bader’s theory of “Atoms in Molecules” (AIM, Figures 5 and 6 and Table S2). We performed calculations for both four-membered and six-membered intermediates, in neutral and protonated forms. We confirmed that the neutral (nonprotonated) six-membered collision complexes (intermediates) are 3× more stable than four-membered structure due to the well-developed network of intermolecular interactions in the six-membered complexes (binding energy (BE) = −14.1 and −14.4 kcal mol^–1^ for six-membered complexes of conformations 1 and 2, respectively, *versus* −4.3 kcal mol^–1^ for four-membered intermediate, Table S1).

**Figure 5:**
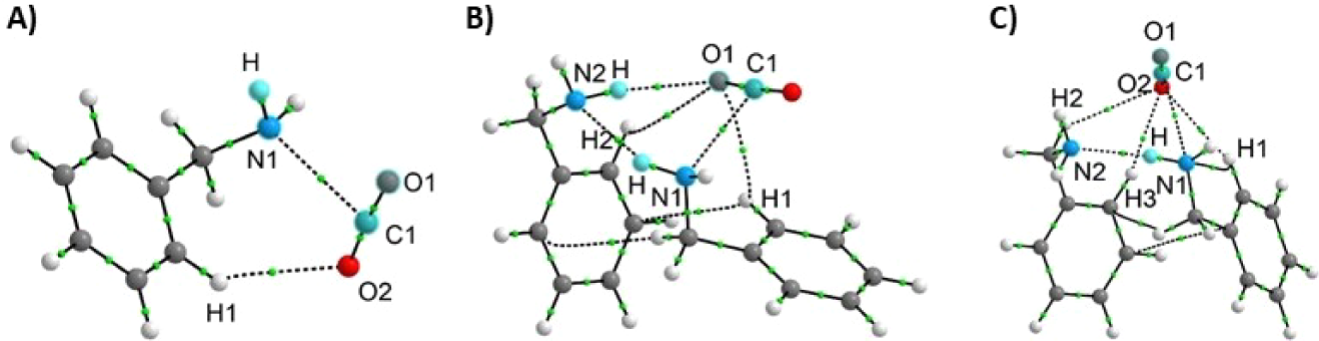
Structures of neutral, nonprotonated four-membered (A) and six-membered collision complexes (B and C) between benzylamine and CO_2_. The structure in (B) represents conformation **1**, and that in (C) represents conformation **2** for the same six-membered complex. The noncovalent interactions (dashed lines) were identified with QTAIM analysis.

**Figure 6:**
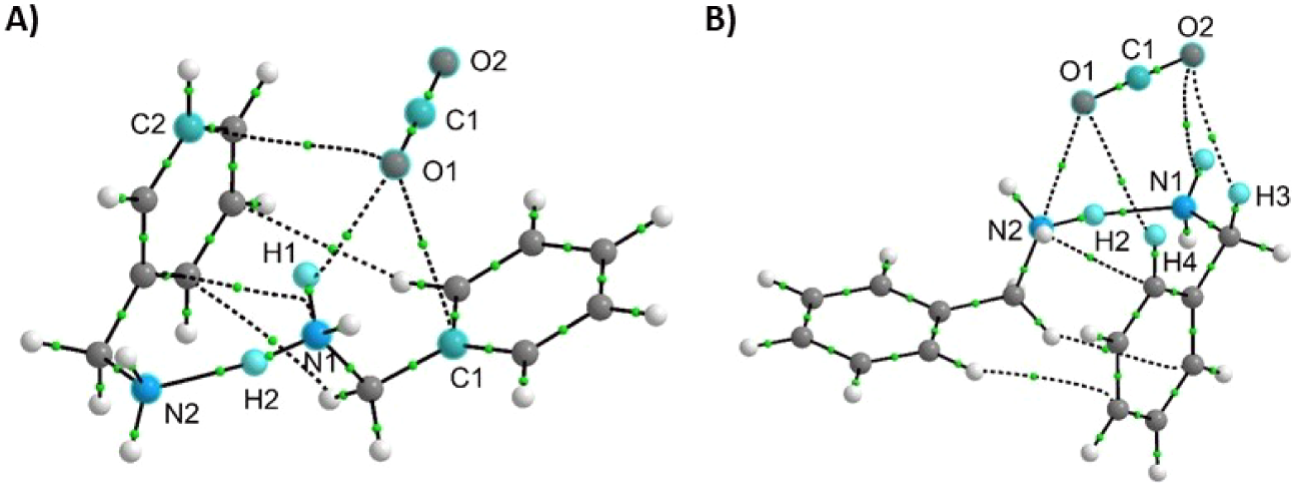
Structure of NH_2_-protonated collision complexes (A) and (B) between two benzylamine molecules and CO_2_. The marked noncovalent interactions (dashed lines) were identified with QTAIM analysis. The structure in (A) represents conformation **1**, and that in (B) represents conformation **2** for the protonated six-membered complex.

The next step in the calculation was to determine the most favorable protonation site for the neutral complexes, where we considered three possibilities: the O atom of the CO_2_ molecule (Table S3), the N atom of the benzylamine molecule (Table S4), and the C atom (in *para* position) of the benzene ring (Table S5). As expected, the NH_2_ group in benzylamine (which has the highest proton affinity) is the most probable site of proton attachment in the intermediate systems. This results in protonated complexes that are most thermodynamically stable for both four- and six-membered ring conformations. Protonation of the N atom of benzylamine did not transform the complexes into the reaction products (i.e., benzylcarbamic/benzylamine salt) during geometry optimizations. This observation is contrary to the protonation of the relatively unfavorable CO_2_ site, which led to the collapse of the complex into new species, [PhCH_2_NHCOOH]­[PhCH_2_NH_3_]^+^ (see Tables S3 and S4 for details).

Like the neutral counterparts, the protonated six-membered conformations are 5× more stable than the four-membered complex (−39.3 and −37.2 kcal mol^–1^ for conformations **1** and **2** *versus* −7.9 kcal mol^–1^ for four-membered one, Table S4). Interestingly, protonation further stabilized the four- and six-membered complexes (i.e., BE for protonated six-membered complex at NH_2_ group is −37.2/–39.3 kcal mol^–1^, while BE for nonprotonated complex is only −14.1/–14.6 kcal mol^–1^, Tables S1, S4). Note that the product [PhCH_2_NHCOOH]­[PhCH_2_NH_3_]^+^ (overall positive) obtained after protonating the six-membered complex at CO_2_ is different from the benzylcarbamic/benzylamine salt [PhCH_2_NHCOO^–^]­[PhCH_2_NH_3_]^+^ (overall neutral). We observed the protonation of the six-membered complex at NH_2_ group to be more stable (by 5.3–12 kcal mol^–1^) than the protonated product obtained during CO_2_ protonation (Tables S3, S4). Also, based on our DFT studies, benzylcarbamic acid/benzylamine salt (same mass as the six-membered intermediate) cannot exist in a gas phase as an ionic species. Instead, this salt undergoes spontaneous intramolecular proton transfer during geometry optimization from NH_3_^+^ group to NHCOO^–^ group to form a complex between benzylcarbamic acid and benzylamine [Ph–CH_2_–NHCOOH]­[Ph–CH_2_–NH_2_], which is stabilized by hydrogen bonding (BE of intramolecular protonation = 134–140 kcal mol^–1^, Table S6). Collectively, these findings suggest that protonation of NH_2_ groups in the three-component six-membered complex can be detected *via* MS in the gas phase (Figure 3A). On the contrary, protonation of the intact benzylcarbamic acid/benzylamine salt is not feasible, indicating its detection by MS is unlikely. Instead, the individual components of this salt are detected independently in our MS experiment, as discussed previously (Figure 2 and S9). See other details in Supporting Information regarding the specific network of interactions detected in the two conformations of the six-membered intermediate shown in Figures 5 and 6.

### Capture Capacity of Different Amines

The formation of the solid in our contained-SESI setup offers an opportunity to directly measure the CO_2_ capture capacity for different amines within a specific time point (we used an optimized 5 min capture time for all amines tested (Figure S11)). For this purpose, CO_2_ gas was exposed to different amines to form carbamic acid salts in a container. By testing different amines including propylamine, butylamine, benzylamine, 3-phenylpropylamine, and a mixture (1:1 v/v) of propylamine/3-phenylpropylamine, we were able to measure their capacity to capture CO_2_ (Figure 7). As the molecular weight of the amine increased, its mass percent of captured CO_2_ or mmol/g capture capacity (Figure S12) decreased. However, the mole percent of CO_2_ capture remained nearly identical. These results are in accordance with the theoretical CO_2_ capture capacity of primary amines (0.5 mol of CO_2_ *per* 1 mol of amine). Unlike other MS-based approaches for evaluating CO_2_ capture capacities of amines, which can estimate only an approximate conversion ratio of amines (because of differences in ionization efficiencies between reactants and products), our method allows collection of products in the container. Capture capacity is evaluated simply by mass difference (before and after CO_2_ addition), providing fast and precise reaction yields.

**Figure 7:**
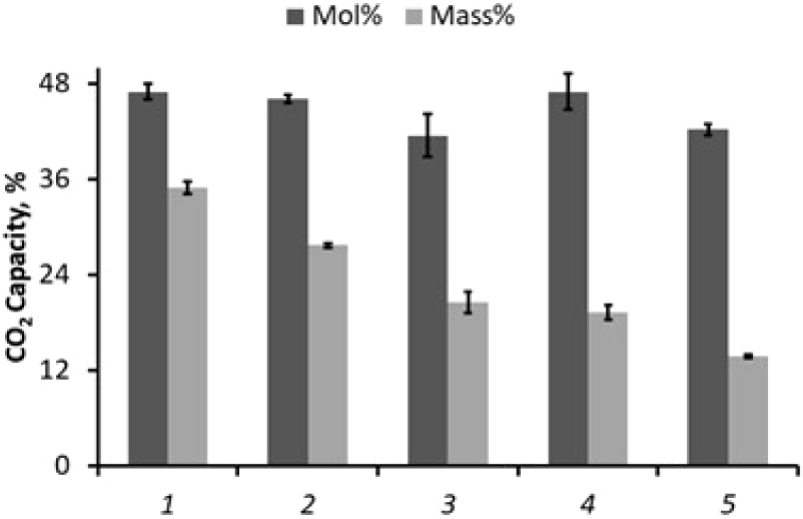
CO_2_ capture capacity for different amines as determined by contained-SESI platform. Amines tested include propylamine (**1**; MW 59 Da), butylamine (**2**; MW 73 Da), propylamine/3-phenylpropylamine (**3**), benzylamine (**4**; MW 107 Da), and 3-phenylpropylamine (**5**; MW 135 Da).

Other critical parameters that can be studied by contained-SESI are related to the energy required for amine regeneration. As discussed earlier, the heat released during CO_2_ capture can be measured simply using a laser thermometer. To determine if this energy corresponds to the melting temperature, the vial containing the crystal powder was heated until the benzylcarbamic acid/benzylamine salt melted. The melting temperature recorded (≈50 °C) in this experiment was the same as the heat released during CO_2_ capture. Note: Teflon vials were used as reaction containers for the heating experiments.

### High-Throughput Analysis

The initial sets of experiments involved *online* monitoring of CO_2_ capture products by MS, as controlled by different modes of operating the contained-SESI source. In this way, we were able to optimize the performance of the platform with ease, while also offering a quantitative way to measure the amount (20 μM) of amine vapor that engages in the CO_2_ capture/reaction. See Supporting Information (Topic 19 and Figures S13–S23) for details on optimized parameters and comparison of various ion sources with the current contained-SESI source. Our contained-SESI source showed superior sensitivity for the detection of CO_2_ capture products compared to previously reported platforms (Figure S22).

So far, for both solidification and *online* MS capture experiments, we have discussed the interaction of a single amine with CO_2_ *per* experiment. However, another important feature of the contained-SESI setup is its ability to enable high-throughput screening, where up to five different amines can be tested for their ability to capture CO_2_ and corresponding products detected in real-time by MS. For this high-throughput experiment, we used a rotating disk that housed five containers, which were in turn attached to the cross Swagelok element of the contained-SESI platform, as illustrated in Figure 8A. As the rotating disk switches position, the headspace vapors of different amines are sampled, one after another, by the N_2_ gas. We used N_2_ nebulizer gas (100 psi) in this experiment to prevent amine solidification. The CO_2_ reactant was captured from ambient air. CO_2_ nebulizer gas can be used if diluted amine solutions are employed. The sampled gas mixture (amine vapor and N_2_) is transferred to the reaction cavity, where charged microdroplets derived from methanol are delivered, creating a liquid film that dissolves the gases into solution and subsequently facilitates their reaction. Reaction products were detected and characterized by MS in real-time. Overall, the amines tested in this high-throughput experiment include benzylamine (vapor pressure (VP) = 8.8 × 10^–2^ kPa), 3-phenylpropylamine (VP = 1.5 × 10^–2^ kPa), butylamine (VP = 12.4 kPa), hexylamine (VP = 1.2 kPa), and propylamine (VP = 41.3 kPa).

**Figure 8:**
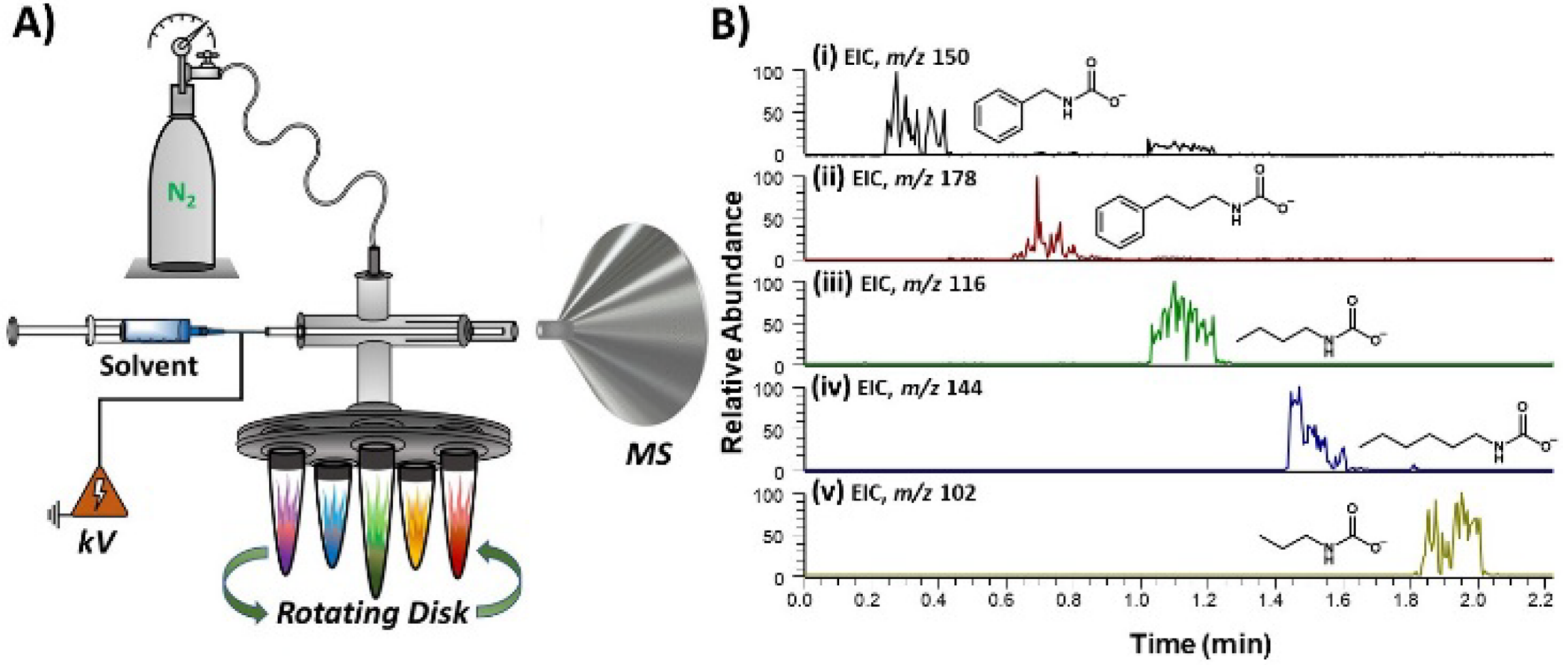
(A) High-throughput contained-SESI MS platform showing a rotating disk to switch between different containers with varying amines: benzylamine, 3-phenylpropylamine, butylamine, hexylamine, and propylamine. (B) Negative-ion mode extracted ion chronograms for deprotonated carbamic acids derived from the real-time interaction of CO_2_ in ambient air with headspace vapors of (i) benzylamine, (ii) 3-phenylpropylamine, (iii) butylamine, (iv) hexylamine, and (v) propylamine.

Figure 8B shows the extracted ion chronograms (XIC) recorded from this high-throughput contained-SESI experiment. To minimize significant carryover effect, the rotating disk was set to switch one amine container after another such that there was 12 s pause interval (i.e., with no amine sampled within this pulsed interval). When analysis was initiated, the amine vapor was sampled for 10 s. Therefore, the analysis of all five amines was accomplished in under 1.7 min. Mass spectra of the different amine-CO_2_ capture products are provided in Figures S24–S28, which display high signal-to-noise ratios, thus showcasing the sensitivity of the platform, even for the capture of CO_2_ from ambient air using amines with appropriate vapor pressures.

### Less Volatile Amines

The use of less volatile amines for CO_2_ capture could have significant industrial significance. Therefore, we tested the potential to sample headspace vapors from less volatile amines: 3-phenylpropylamine (liquid) and dodecylamine (DDA, solid, VP = 1.1 × 10^–3^ kPa). Both amines were successfully detected as protonated species in the positive-ion mode. Detection sensitivity was significantly improved by diluting/dissolving the amines in a more volatile methanol (16.9 kPa) solvent (10:1, v/v, amine to methanol for liquid amine; 10:1, w/w, amine to methanol for solid amine). For example, the absolute ion intensity of 3-phenylpropylcarbamate increased by about 30X (Figure S29) and of protonated DDA by about 150X (CO_2_ capture product was not detected at this low VP, Figure S30). Surprisingly, acetone (30.8 kPa) did not affect the volatility of DDA, despite having higher vapor pressure and solubility. This effect may be attributed to the limited ability of acetone to form hydrogen bonding compared with methanol. This limited chemical interaction in turn limits the ability of acetone to lift up DDA into the air for subsequent headspace vapor analysis by the contained-SESI platform. To further improve the detection of less volatile amines as well as their CO_2_ capture capacity, future studies will include controlled heating of the container, the reaction cavity thin films, and reaction microdroplets.

Additionally, instead of sampling headspace vapor, the amine solution can be sprayed directly into the reaction cavity for evaluation of CO_2_ capture abilities (Type IV operation mode). For example, no CO_2_ capture product is detected for N,N-dibutyl-1,3-propanediamine (DBPA, estimated VP = 1.9 × 10^– 3^ kPa)^32^ when a 10 mM solution is analyzed by conventional ESI (positive ion mode) with N_2_ nebulizer gas, which is in agreement with a previous report.^13^ However, significant CO_2_ capture product is detected when the reaction cavity is introduced into the contained-ESI source. An absolute ion intensity of 10^6^ signal was recorded for the product at *m*/*z* 231 when the DBPA solution (10 mM) was sprayed with the contained-ESI source having 8 mm cavity size (Figure S31).

## Conclusions

In summary, the current study presents experimental and computational DFT data confirming that the six-membered intermediate (so-called 1,5-zwitterion) formed between two molecules of amine and one molecule of CO_2_ is more stable than the corresponding four-membered (1,3-zwitterion) intermediate, which is formed between one molecule each of the amine and CO_2_. The specific evidence involves (1) the detection of [2 M + CO_2_ + H]^+^ species in the positive-ion mode MS but not [M + CO_2_ + H]^+^ species, and (2) DFT data showing more stable neutral and protonated six-membered complexes than the corresponding four-membered intermediates.

The chemical composition of the six-membered intermediate directly correlates with the final product, carbamic acid/amine salt, expected for CO_2_-amine capture chemistry, proving a potential concerted reaction as suggested by others. We hypothesize, however, that the detection of the carbamic acid/amine salt in the gas phase is not likely, since the protonation of this complex, and hence neutralization of the anion counterpart, will lead to instantaneous weakening of the ionic bonds holding the salt together. Other pathways for disassembly of the salt may involve energetically favorable intramolecular proton transfer from the cationic component to the anionic part of the salt as seen in our DFT calculations. Instead, we propose that the neutral six-membered intermediate must be formed prior to the formation of the carbamic acid/amine salt. Therefore, the species detected at *m*/*z* 259 is assigned to the protonated six-membered intermediate, the existence of which is proven by DFT calculations.

The contained secondary electrospray experimental setup allowed the headspace vapor from primary amines, with varying vapor pressures, to be sampled and reacted with CO_2_ gas. The main novel feature of this SESI experiment is the inclusion of the reaction cavity at the outlet of the source, which enabled the creation of super-reactive droplets/thin film for sensitive monitoring of reaction intermediates and products by mass spectrometry. The ability to sample headspace vapors enabled the development of a high-throughput experimentation in which the analysis of five different amines (vapor pressure >10^–2^ kPa) was conducted individually with CO_2_ gas in under 2 min.

The containment feature of our SESI source not only enables sampling of amine headspace vapor but also allows CO_2_ gas to directly interact with liquid-phase amine. This provided an opportunity to isolate crystalline forms of the carbamic acid/amine salt for characterization by Fourier transform infrared spectroscopy. Overall, the fast response time, high sensitivity, and ease of operating the contained-SESI platform enable product synthesis on the milligram scale, real-time reaction monitoring in terms of mass-to-charge ratio, kinetics, thermodynamics, reaction yield, and CO_2_ capture capacity. We believe this platform opens a window to study the capture mechanism of new materials as well as to measure the capture capacity in a straightforward manner and in a single experiment.

## Supplementary Material



## Notes

Identification of carbamate products from the reaction of different amines with CO_2_ (Figure S1); high-resolution Orbitrap data (Figure S2); analysis of synthesized butylcarbamic acid/butylamine salt (Figure S3); optimization of spray solvent composition and capillary inlet temperature (Figure S4); effect of RF voltage on the fragmentation of protonated benzylamine (Figures S5, S6); solid-state FTIR analysis of the white powder product (Figures S7, S8); analysis of synthesized benzylcarbamic acid/benzylamine salt (Figure S9); kinetics studies for product formation using contained-SESI platform (Figure S10); DFT studies (Tables S1–S7); determination of CO_2_ capture capacity of different amines by contained-SESI (Figure S11); influence of molecular weights of amines on their CO_2_ capture capacity by contained-SESI (Figure S12); cavity size effect on product formation (Figure S13); solvent and temperature effects on the benzylamine vapor fragmentation by contained-SESI (Figure S14); optimizations of solvent flow rate, gradient flow rate, and spray voltage for butylcarbamate product formation in contained-SESI (Figures S15–S17); spray voltage effect in contained-SESI (Figures S18, S19); N_2_ gas pressure optimization for butylcarbamate product formation in contained-SESI (Figure S20); N_2_ or CO_2_ nebulizer gas effects on benzylcarbamate product formation in contained-SESI (Figure S21); comparison of different ionization techniques on benzylcarbamate formation (Figure S22); optimization of *online* CO_2_ capture; full mass spectra from high-throughput analysis by contained-SESI (Figures S24–S28); effect of solvent addition on the formation of 3-phenylpropylcarbamate by contained-SESI (Figure S29); solvent addition effect on the vapor pressure of dodecylamine by contained-SESI (Figure S30); effect of cavity size on diamine carbamate product formation by contained-SESI (Figure S31) (PDF)
