# Supplementary material for: A Six-Membered Concerted Mechanism for CO2 Capture by Amines Studied under Charged Microdroplet Reaction Conditions
Source: Anal Chem. 2026 Apr 22;98(17):12790–801. doi: 10.1021/acs.analchem.6c00175 (PMC13150804; doi:10.1021/acs.analchem.6c00175)
Supplement: Supplementary file 1 [file ac6c00175_si_001.pdf]

SUPPORTING INFORMATION

A Six-Membered Concerted Mechanism for CO_2 Capture by Amines Studied under Charged Microdroplet Reaction Conditions

Taghi Sahraeian,[∇,†] Dmytro S. Kulyk,[∇,†] Ayesha Seth,[†] Glib V. Baryshnikov,[§] and Abraham K. Badu-Tawiah*[,†]

[†]Department of Chemistry and Biochemistry, The Ohio State University, Columbus OH, 43210

[§]Laboratory of Organic Electronics, Department of Science and Technology, Linköping University, SE-60174 Norrköping, Sweden

[∇]These authors contributed equally

*Corresponding Author:

Prof. Abraham Badu-Tawiah: Tel.: (614) 929-4276, and Fax: (614) 929-1685

Email: badu-tawiah.1@osu.edu

Topic	Title of Topic	Page
**Topic 1** (Figure S1)	Identification of carbamate products from reaction of different amines with CO_2	S4
**Topic 2** (Figure S2)	High resolution Orbitrap data	S5
**Topic 3** (Figure S3)	Analysis of synthesized butylcarbamic acid/butylamine salt	S6
**Topic 4** (Figure S4)	Optimization of spray solvent composition and capillary inlet temperature	S7
**Topic 5** (Figure S5, S6)	Effect of RF voltage on fragmentation of the protonated benzylamine	S8
**Topic 6** (Figure S7, S8)	Solid state FTIR analysis of the white powder product	S9
**Topic 7** (Figure S9)	Analysis of synthesized benzylcarbamic acid/benzylamine salt	S10
**Topic 8** (Figure S10)	Kinetics studies for product formation using contained-SESI platform	S11
**Topic 9** (Tables S1−S7)	DFT Studies	S12
**Topic 10** (Figure S11)	Determination of CO_2 capture capacity of different amines by contained-SESI	S19
**Topic 11** (Figure S12)	Influence of molecular weights of amines on their CO_2 capture capacity by contained-SESI	S20
**Topic 12** (Figure S13)	Cavity size effect on the product formation	S21
**Topic 13** (Figure S14)	Solvent and temperature effect on the benzylamine vapor fragmentation by contained-SESI	S22
**Topic 14** (Figures S15−S17)	Optimizations of solvent flowrate, gradient flowrate, and spray voltage for butylcarbamate product formation in contained-SESI	S23
**Topic 15** (Figure S18, S19)	Spray voltage effect in contained-SESI	S25
**Topic 16** (Figure S20)	N_2 gas pressure optimization for butylcarbamate product formation in contained-SESI	S26
**Topic 17** (Figure S21)	N_2 or CO_2 nebulizer gas effects on benzylcarbamate product formation in contained-SESI	S27
**Topic 18** (Figure S22, S23)	Comparison of different ionization techniques on benzylcarbamate formation	S28
**Topic 19**	Optimization of *online* CO_2 capture	S29
**Topic 20** (Figures S24−S28)	Full mass spectra from high-throughput analysis by contained-SESI	S31

**Topic 21** (Figure S29)	Effect of solvent addition on the formation of 3-phenylpropylcarbamate by contained-SESI	S34
**Topic 22** (Figure S30)	Solvent addition effect on the vapor pressure of dodecylamine by contained-SESI	S35
**Topic 23** (Figure S31)	Effect of cavity size on diamine carbamate product formation by contained-SESI	S36
**Topic 24**	References	S37

1. Identification of Carbamate Products from Reaction of Different Amines with CO_2

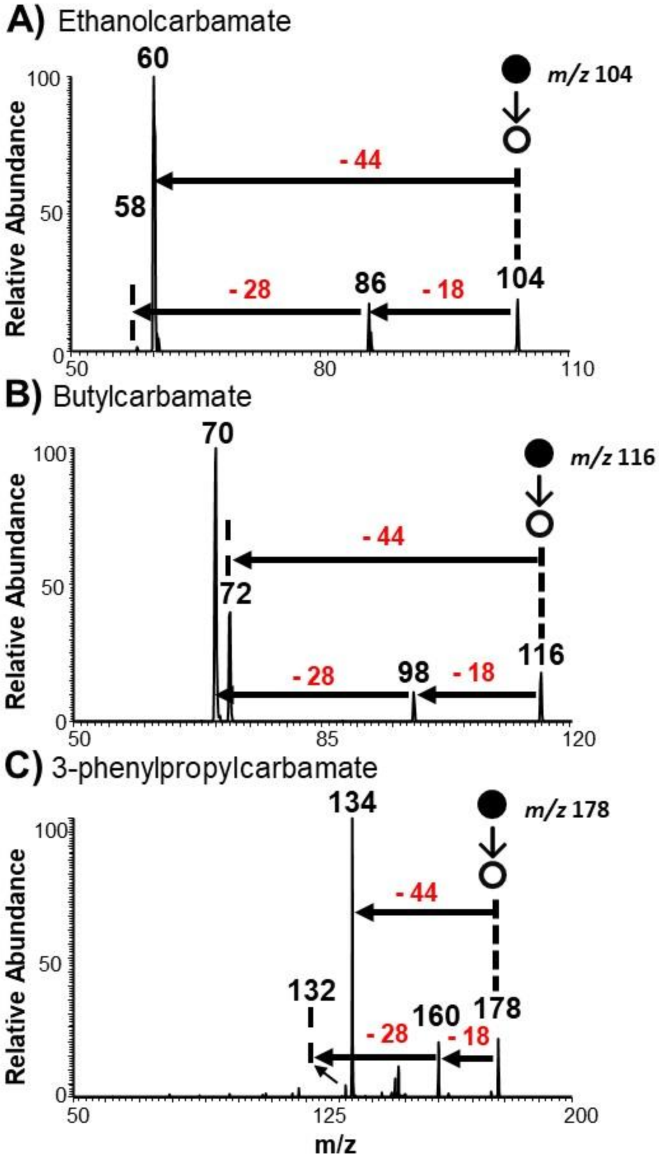

Figure S1. Tandem mass spectra of carbamate products from the reaction of different amines with CO_2 by contained secondary ESI which generate the same fragmentation patterns: **A)** ethanolcarbamate, **B)** butylcarbamate, and **C)** 3-phenylpropylcarbamate.

2. High Resolution Orbitrap Data

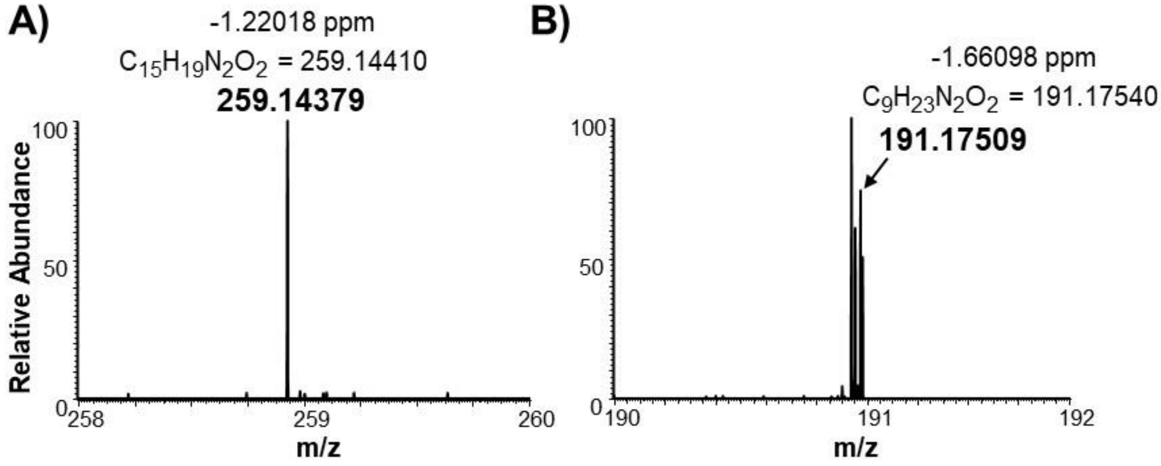

Figure S2. High resolution Orbitrap mass spectra for **A)** benzylcarbamic acid/benzylamine salt and **B)** butylcarbamic acid/butylamine salt dissolved in MeOH. Elemental composition analysis assigned the *m/z* 259 and 191 peaks to the $[2M + CO_2 + H]^+$ species, yielding mass accuracies of 1.22 ppm and 1.66 ppm, respectively.

3. Analysis of Synthesized Butylcarbamic Acid/Butylamine Salt

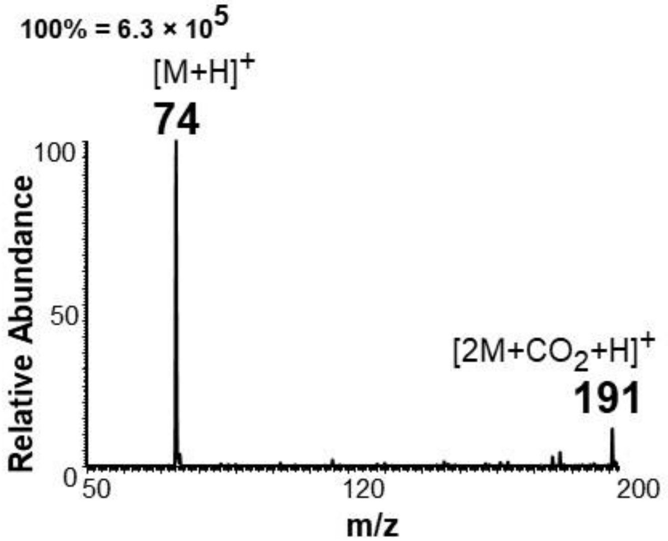

Figure S3. Synthesized solid butylcarbamic acid/butylamine salt was dissolved in MeOH and analyzed by contained-SESI MS in ESI mode.

4. Optimization of Spray Solvent Composition and Capillary Inlet Temperature

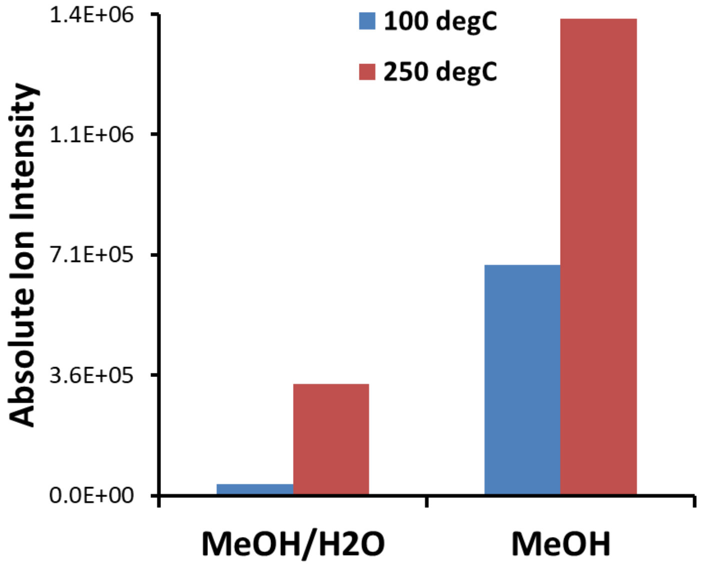

Figure S4. The bar graph demonstrating the effect of spray solvent composition and mass spectrometer capillary inlet temperature on analyte (benzylamine) signal intensity in positive ion mode. The results show that pure MeOH is better than the mixture (1:1 v/v) of MeOH/H_2O and inlet temperature of 250 °C produces higher absolute intensity.

5. Effect of RF Voltage on Fragmentation of the Protonated Benzylamine

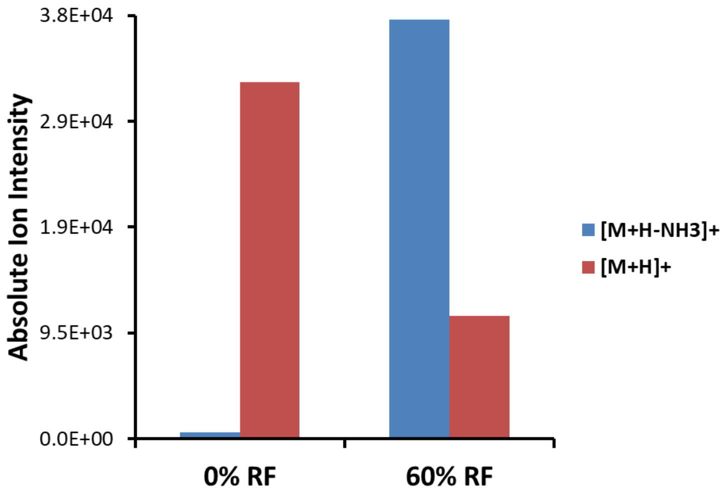

Figure S5. Bar graph showing the effect of RF voltage on the fragmentation of protonated benzylamine peak in positive ion mode. The RF voltage causes the *m/z* 108 fragments to *m/z* 91, however, a 0 RF voltage preserves the total intensity of the *m/z* at 108.

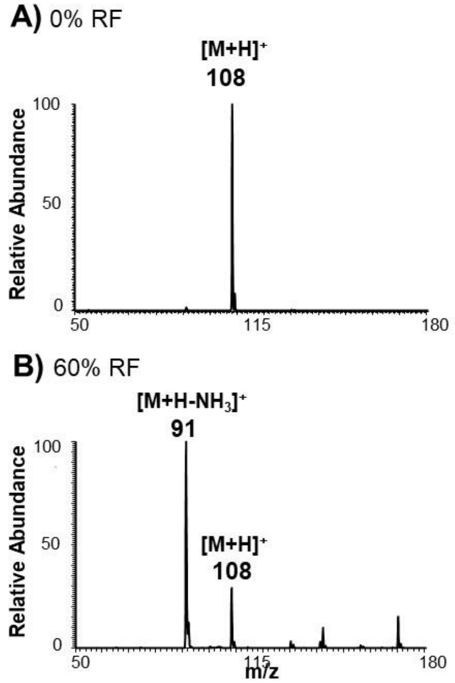

Figure S6. Positive mode full mass spectra showing the effect of RF voltage on the fragmentation of protonated benzylamine product peak for contained-SESI. **A)** The 0% RF voltage shows no fragmentation of peak at *m/z* 108 while **B)** 60% RF voltage causes some of the *m/z* 108 fragments to *m/z* 91.

6. Solid State FTIR Analysis of the White Powder Product

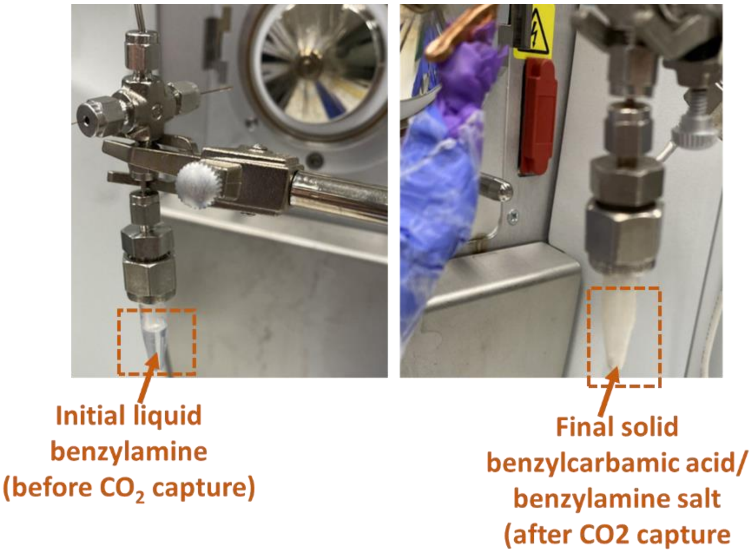

Figure S7. Formation of benzylcarbamic acid/benzylamine salt.

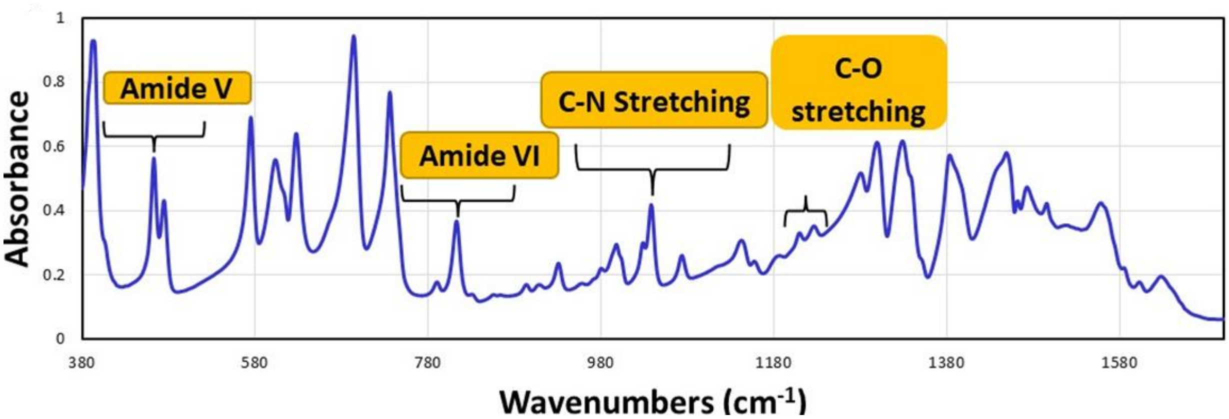

Figure S8. The solid state FTIR spectrophotogram for white powder product benzylcarbamic acid/benzylamine salt produced from the reaction of benzylamine vapor with CO_2 gas using contained-SESI platform.

7. Analysis of Synthesized Benzylcarbamic Acid/Benzylamine Salt

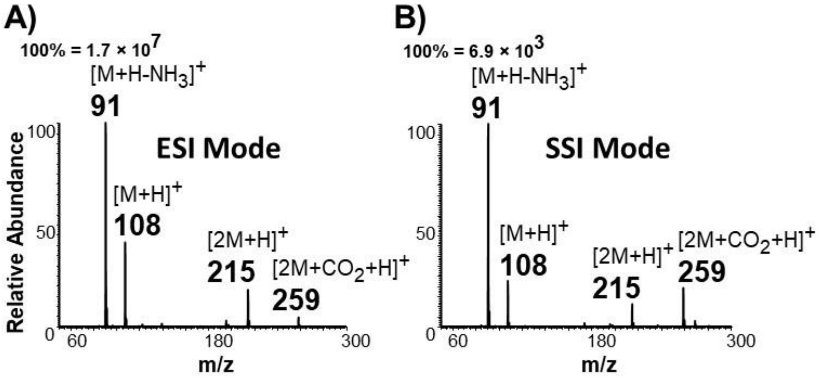

Figure S9. Synthesized solid benzylcarbamic acid/benzylamine salt was dissolved in MeOH and analyzed by contained-SESI MS in **A)** ESI mode (5 kV spray voltage, no reaction cavity) and **B)** SSI mode (no voltage, no reaction cavity). Protonated amine ($[M + H]^+$, *m/z* 108) and protonated 6-membered intermediate complex $[2M + CO_2 + H]^+$ as well as their collision fragments (NH_3 loss for the amine: $[M + H - NH_3]^+$, *m/z* 91; and CO_2 loss for the complex: $[2M + H]^+$, *m/z* 215) were detected by both ESI and SSI modes. SSI mode appears to be softer ionization method to preserve better the complex without its significant defragmentation.

8. Kinetics Studies for Product Formation using Contained-SESI Platform

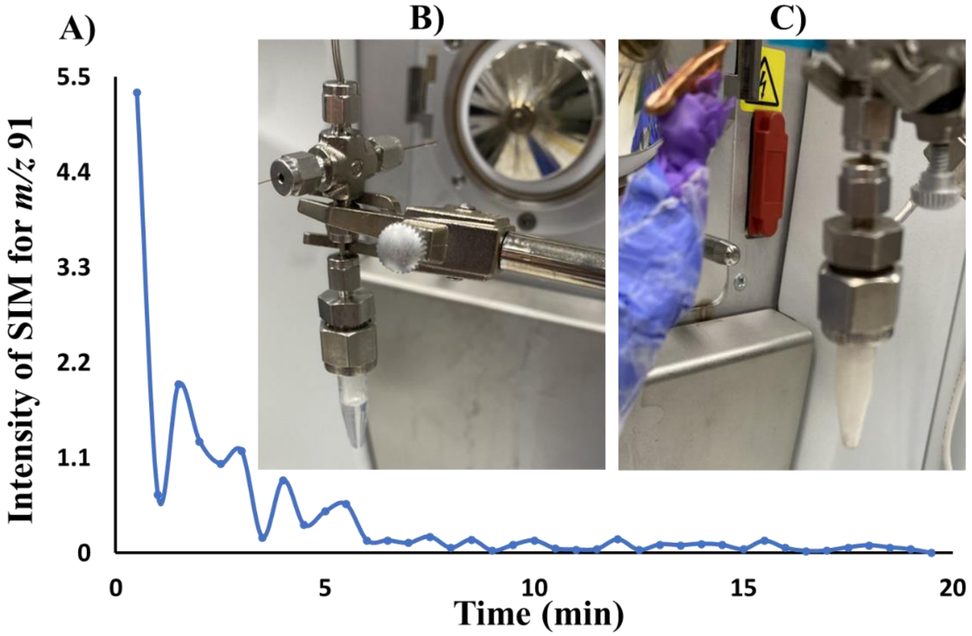

Figure S10. A) Time measurement for product formation from the reaction between benzylamine and CO_2 by monitoring the disappearance of the SIM at *m/z* 91 from the protonated reagent benzylamine with *m/z* 108 by contained-SESI setup **B)** containing liquid benzylamine before reaction starts and **C)** after reaction completion converting the liquid benzylamine into a white powder of benzylcarbamic acid/benzylamine salt.

9. DFT Studies

The geometry optimization of the reagent, products and all intermediates along the transformation pathway was caried out by using B3LYP functional,[1,2] 6-311g(d,p)[3,4] basis, and empirical dispersion at GD3[5] level. All calculations were performed in a gas phase approximation. The energetics along the transformation mechanism were calculated accounting for zero-point energy (ZPE) correction. The simulations were carried out by using Gaussian16 software.[6] Analysis of electron density distribution for the studied collision complexes was performed by using Bader's theory of "Atoms in Molecules" (AIM)[7–9] implemented in AIMAll (version 19.10.12) software.[10]

Non-protonated complexes. We started the simulations from optimization of neutral non-protonated complexes following the 4-membered and 6-membered mechanisms as well as the simulations corresponded to only benzylamine molecule and CO_2 molecule (**Table S1**). We obtained the complexes with clear intermolecular interactions between CO_2 and benzylamine. Although the distances between molecules are considerable, we can observe the bending of CO_2 molecule by approximately 5° for 4-membered intermediate and 4-6° for 6-membered intermediate. These were simulated in two different conformations. Calculations of binding energy (BE) (with consideration to zero-point energy correction) indicates that the formation of 6-membered collision complexes in both conformations is more favorable than the formation of the 4-membered complex (BE = −14.1 and −14.4 kcal mole^{-1} for 6-membered complexes of conformations ***1*** and ***2***, respectively, *versus* −4.3 for kcal mole^{-1} for 4-membered ring, **Table S1**).

Table S1. Optimized structures of 4-membered and 6-membered complexes (conformations ***1*** and ***2***) of benzylamine and CO_2. The numbers in the conformations represent selected interatomic distances (Å), ω – vibration frequencies (cm^{-1}).

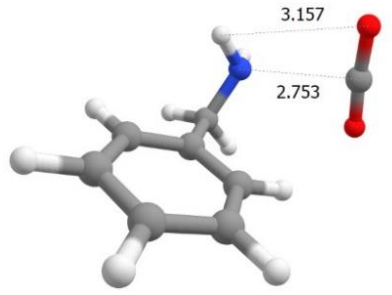	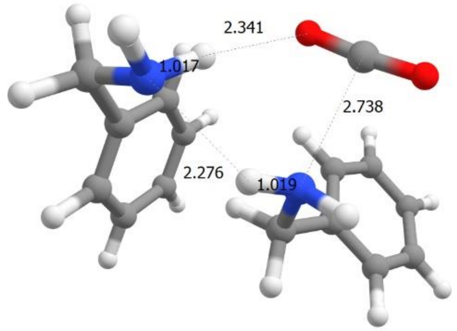	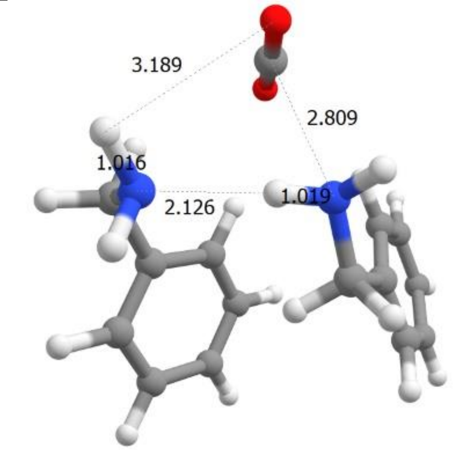
**4-Membered Complex (ω_1=12 cm^{-1})**	**6-Membered Complex, Conform. *1* (ω_1=13 cm^{-1})**	**6-Membered Complex, Conform. *2* (ω_1=8 cm^{-1})**
$E_{tot} + E_{ZPE}$ = -515.498370 a.u.	$E_{tot} + E_{ZPE}$ = -842.374583 a.u.	$E_{tot} + E_{ZPE}$ = -842.375448 a.u.
BE = -4.3 kcal mole^{-1}	BE = -14.1 kcal mole^{-1}	BE = -14.6 kcal mole^{-1}
Benzylamine	$E_{tot} + E_{ZPE}$ = -326.860612 a.u. (ω_1=53 cm^{-1})	
CO_2	$E_{tot} + E_{ZPE}$ = -188.630940 a.u. (ω_1=654 cm^{-1})	

After structural optimization, we performed the analysis of electronic density distribution for all three collision complexes using Bader's theory of "Atoms in Molecules" (AIM).[7-9] The resulting figures showing the detected non-covalent interactions (dashed lines) are presented in **Figure 1** (main manuscript). The key characteristics of the bond critical points (BCP, green dots in **Figure 1**) for non-covalent interactions are summarized in **Table S2**.

From **Table S2**, we can see that all the detected non-covalent interactions are characterized by positive values of electron energy density h(r) and electron density Laplacian $\nabla^2\rho(r)$ that classify them as typical interactions of closed shell with small covalency character. Indeed, delocalization index (DI) never exceed 0.1, meaning that electron density is only slightly shared in the interatomic space of interacting atoms. This is typical for weak hydrogen bonds and van-der-Waals interactions. Positive electron energy density h(r) originates from prevailing kinetic energy density g(r) over the potential energy density v(r). The latter was used for estimation of non-covalent interaction energy (E) by using simple empirical relation by Espinosa and co-authors: E=0.5v(r).[11] The total energy of non-covalent interactions correlates well with the calculated binding energies (BE) of studied collision complexes. This means that the 6-membered collision complexes are more stable than 4-membered structure due to the well-developed network of intermolecular interactions in 6-membered complexes. Note: some C---H intermolecular contact are omitted in **Table S2**, but they also additionally contribute to the total binding energy of the complex.

Comparing the 6-membered conformations **(B)** and **(C)**, we can observe that conformation **(B)** represents the genuine 6-membered coupling scheme *via* three intermolecular interactions C1---N1, N1H---N2 and O1---H, while conformation **(C)** and 4-membered complex **(A)** do not really show the intermolecular coupling corresponding to the formation of 6- and 4-membered coupling.

Table S2. Bond lengths (*d*), energies (*E*), and the topological characteristics of the electron density distribution in the critical point (3, –1) for non-covalent interactions in the three collision complexes between benzylamine and CO_2.

Bond	d, Å	ρ(r), $e\cdot a_0^{-3}$	v(r), *au*	g(r), *au*	h(r), *au*	$\nabla^2\rho(r)$, $e\cdot a_0^{-5}$	ε	DI	E, kcal mole^{-1}
**4-Membered Complex**									
C1---N1	2.753	0.0165	-0.0108	+0.0117	0.0008	0.0500	0.67	0.047	3.38
O2---H1	2.513	0.0083	-0.0049	+0.0058	0.0009	0.0268	0.06	0.033	1.53
**6-Membered Complex (Conformation *1*)**									
C1---N1	2.738	0.0172	-0.0111	0.0119	0.0008	0.0504	0.62	0.050	-3.48
N2---HN1	2.276	0.0160	-0.0093	0.0111	0.0018	0.0519	0.28	0.062	-2.92
O1---N2H	2.341	0.0101	-0.0060	0.0073	0.0013	0.0345	0.03	0.034	-1.88
O1---H1	2.799	0.0056	-0.0031	0.0041	0.0010	0.0204	1.72	0.018	-0.97
O1---H2	2.932	0.0039	-0.0022	0.0030	0.0008	0.0148	0.28	0.012	-0.69
**6-Membered Complex (Conformation *2*)**									
C1---N1	2.809	0.0135	-0.0086	0.0099	0.0013	0.0445	0.65	0.038	-2.70
N2---HN1	2.126	0.0226	-0.0140	0.0154	0.0014	0.0672	0.04	0.084	-4.39
O2---H2	2.810	0.0050	-0.0029	0.0035	0.0006	0.0165	0.22	0.019	-0.91
O2---H1	2.639	0.0061	-0.0036	0.0042	0.0006	0.0196	0.16	0.022	-1.13
O2---H3	2.510	0.0077	-0.0046	0.0055	0.0009	0.0252	0.04	0.028	-1.44

Protonated complexes. Having confirmed the existence of the six-membered intermediate and its high stability compared with four-membered intermediate for the neutral species, we considered the protonated complexes of the same systems presented in **Table S1** (optimized complexes were protonated

and optimized further). Here, we defined three protonation sites: Oxygen atom of CO_2 molecule, Nitrogen atom of NH_2 group, Carbon atom in *para* position of benzene ring. Moreover, Nitrogen atom of amide group of the synthesized product $[Ph\text{-}CH_2\text{-}NH\text{-}COO]^{-}[Ph\text{-}CH_2\text{-}NH_3]^{+}$ was tested too for possible protonation event. These are discussed in turn below.

1) *Oxygen atom of CO_2 molecule.* We first considered protonation at the Oxygen in CO_2. In all cases for 4- and 6-membered rings, the complex spontaneously transformed into different protonated products (**Table S3**). In the case of 4-membered ring, it transformed into $[Ph\text{-}CH_2\text{-}NH_2\text{-}COOH]^{+}$ compound (H^+ is attached to the COO^- group, *m/z* 152 was not detected by MS). In the case of conformations ***1*** and ***2*** of 6-membered complex, the product is the complex $[Ph\text{-}CH_2\text{-}NH\text{-}COOH][Ph\text{-}CH_2\text{-}NH_3]^{+}$ and there is a short H-bond HOCO---HNH_2 between complex counterparts. Energetically, the complexes with protonated COO^- group is very favourable because of the new C-N covalent bond is formed.

Table S3. Optimized structures of protonated reaction products for 4-membered and 6-membered (conformations ***1*** and ***2***) complexes of benzylamine and CO_2. The numbers represent selected interatomic distances (Å), ω – vibration frequencies (cm^{-1}). The protonation cite was the Oxygen atom of CO_2 molecule. The observed products were $[Ph\text{-}CH_2\text{-}NH_2\text{-}COOH]^{+}$ for 4-membered ring complex and $[Ph\text{-}CH_2\text{-}NH\text{-}COOH][Ph\text{-}CH_2\text{-}NH_3]^{+}$ for 6-membered ring complex.

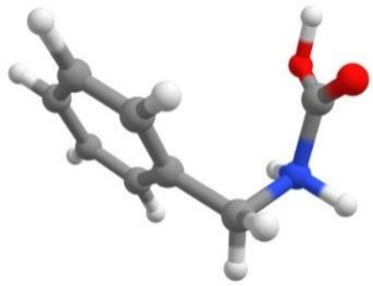	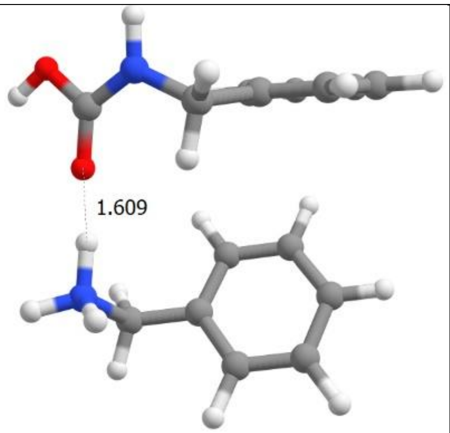	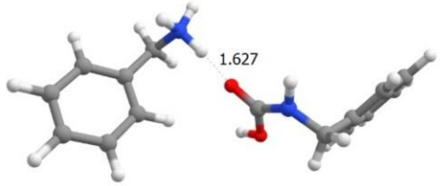
**4-Membered Complex (ω_1=35 cm^{-1})**	**6-Membered Complex, Conform. *1* (ω_1=17 cm^{-1})**	**6-Membered Complex, Conform. *2* (ω_1=6 cm^{-1})**
$E_{tot} + E_{ZPE}$ = -515.814859 a.u.	$E_{tot} + E_{ZPE}$ = -842.759425 a.u.	$E_{tot} + E_{ZPE}$ = -842.752104 a.u.
BE = -75.6 kcal $mole^{-1}$	BE = -128.4 kcal $mole^{-1}$	BE = -123.8 kcal $mole^{-1}$
Benzylamine	$E_{tot} + E_{ZPE}$ = -326.860612 a.u. (ω_1=53 cm^{-1})	
CO_2H^+	$E_{tot} + E_{ZPE}$ = -188.833610 a.u. (ω_1=538 cm^{-1})	

2) *Nitrogen atom of benzylamine molecule.* Now we consider protonation of the complexes at the Nitrogen atom of the benzylamine. In case of 4-membered collision complex, the binding between protonated benzylamine ($PhCH_2NH_3^+$) and CO_2 is realized *via* the single binding mode of short H-bond H_2NH---OCO (1.89 Å). The binding energy is relatively small in respect to the $PhCH_2NH_3^+$ and CO_2 references (**Table S4**). At the same time, the 6-membered collision complexes are much

more stable compared with 4-membered complex (-39.3 and -37.2 kcal mole^{-1} for conformations ***1*** and ***2*** *vs*. -7.9 kcal mole^{-1} for 4-membered one). They are stabilized by a number of intermolecular interactions. For conformation ***1*** the initial 6-membered coupling scheme is destroyed after protonation and CO_2 molecule is oriented similarly to the 4-membered case with quite short H-bond H_2NH---OCO (2.31 Å), while the non-protonated benzylamine forms strong H_2N---HNH_2 bonds with $PhCH_2NH_3^+$ (1.733 Å). In case of conformation ***2*** we can observe the CO_2 binding to both NH_2 and NH_3^+ groups which are additionally coupled *via* short H_2N---HNH_2^+ bond (1.762 Å). For both conformations the H_2N---HNH_2^+ bond can be classified as intermediate type interaction within QTAIM formalism ($\nabla^2\rho(r)>0$, $h(r)<0$) with considerable covalency (DI is near 0.2) and thus high energy (13−14 kcal mole^{-1}).

Table S4. Optimized structures of protonated 4-membered and 6-membered complexes (conformations ***1*** and ***2***) of benzylamine and CO_2. The numbers represent selected interatomic distances (Å), ω – vibration frequencies (cm^{-1}). The protonation cite was the Nitrogen atom of benzylamine molecule. No reaction was observed.

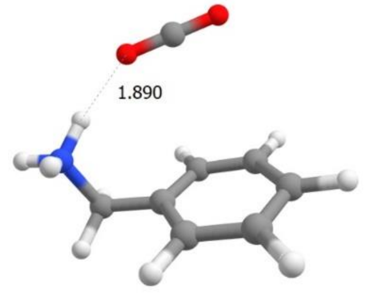

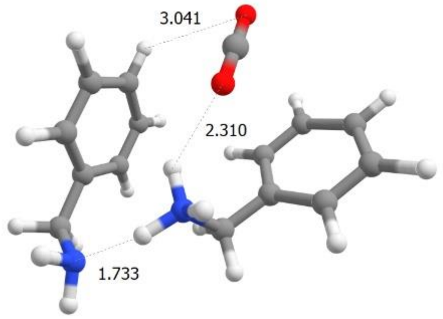

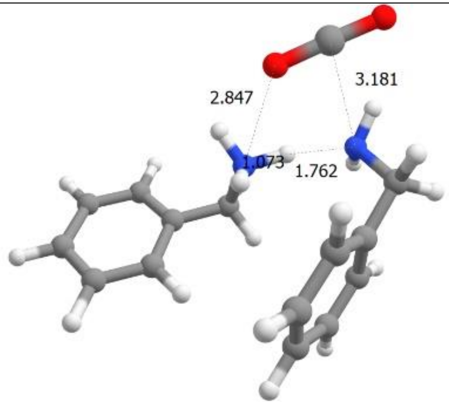

**4-Membered Complex (ω_1=17 cm^{-1})**	**6-Membered Complex, Conform. *1* (ω_1= 20 cm^{-1})**	**6-Membered Complex, Conform. *2* (ω_1= 12 cm^{-1})**
$E_{tot} + E_{ZPE}$ = -515.860488 a.u.	$E_{tot} + E_{ZPE}$ = -842.771142 a.u.	$E_{tot} + E_{ZPE}$ = -842.767929 a.u.
BE = -7.9 kcal mole^{-1}	BE = -39.3 kcal mole^{-1}	BE = -37.2 kcal mole^{-1}

[BenzylamineH]$^+$ ($PhCH_2NH_3^+$)	$E_{tot} + E_{ZPE}$ = -327.217075 a.u. (ω_1=68 cm^{-1})
Benzylamine	$E_{tot} + E_{ZPE}$ = -326.860612 a.u. (ω_1=53 cm^{-1})
CO_2	$E_{tot} + E_{ZPE}$ = -188.630940 a.u. (ω_1=654 cm^{-1})

C atom (in para position) of benzene ring. The protonation of benzene ring doesn't strongly disturb the coupling model between CO_2 and two amine groups (**Table S5**). We can distinguish the 4-membered and 6-membered binding schemes for all considered collision complexes. Similarly to the case of NH_2 protonation, the binding energy of 4-membered scheme is rather small (-3.5 kcal mole^{-1}), while 6-membered schemes for conformations ***1*** and ***2*** are almost equivalent energetically and much more stable than 4-membered one. However, comparing the analogical complexes but different protonation sites, one can find that benzene ring is most unfavourable protonation site (total energies are the smallest compared to CO_2 and NH_2 protonation).

Table S5. Optimized structures of protonated 4-membered and 6-membered complexes (conformations ***1*** and ***2***) of benzylamine and CO_2. The numbers represent selected interatomic distances (Å), ω – vibration frequencies (cm^{-1}). The protonation cite was the Carbon atom (in *para* position) of benzene ring. No reaction was observed.

<table>
<tr><td>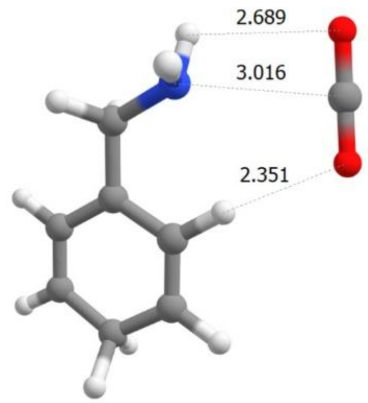
</td><td>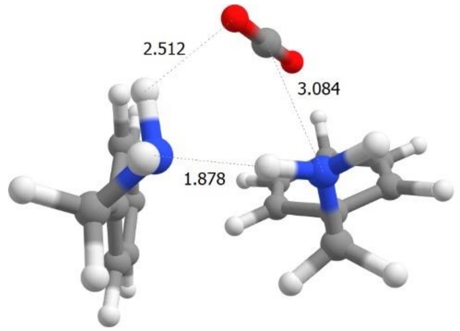
</td><td>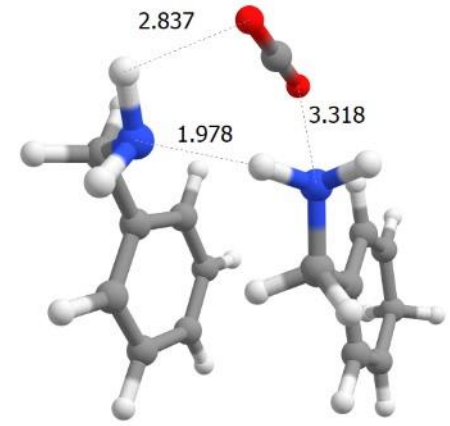
</td></tr>
<tr><td>4-Membered Complex (ω_1= 20 cm^{-1})</td><td>6-Membered Complex, Conform. 1 (ω_1= 18 cm^{-1})</td><td>6-Membered Complex, Conform. 2 (ω_1= 28 cm^{-1})</td></tr>
<tr><td>$E_{tot} + E_{ZPE}$ = -515.808879 a.u.</td><td>$E_{tot} + E_{ZPE}$ = -842.700870 a.u.</td><td>$E_{tot} + E_{ZPE}$ = -842.702413 a.u.</td></tr>
<tr><td>BE = -3.5 kcal $mole^{-1}$</td><td>BE = -23.2 kcal $mole^{-1}$</td><td>BE = -24.1 kcal $mole^{-1}$</td></tr>
<tr><td><u>[BenzylamineH]$^+$ ($C_6H_6CH_2NH_2^+$)</u></td><td colspan="2">$E_{tot} + E_{ZPE}$ = -327.172388 a.u.
(ω_1=81 cm^{-1})</td></tr>
<tr><td><u>Benzylamine</u></td><td colspan="2">$E_{tot} + E_{ZPE}$ = -326.860612 a.u.
(ω_1=53 cm^{-1})</td></tr>
<tr><td><u>CO_2</u></td><td colspan="2">$E_{tot} + E_{ZPE}$ = -188.630940 a.u.
(ω_1=654 cm^{-1})</td></tr>
</table>

3) *N atom of amide group.* The protonation of amide group (-NH-) in the salt product [Ph-CH_2-NH-COO]$^-$ [Ph-CH_2-NH_3]$^+$ during DFT simulation leads to decomposition of the benzylcarbamic acid/benzylamine salt to give initial reagents: benzylamine ($PhCH_2NH_2$), protonated benzylamine ($PhCH_2NH_3^+$), and CO_2, so this is a highly improbable site for protonation.

Conclusion remarks. Among the three sites considered for protonation, we can conclude that protonation of NH_2 group in benzylamine (which has the highest proton affinity) is most probable way of proton attachment in the system. This results complexes that are most thermodynamically stable for both 4- and 6-membered conformations. However, the 6-membered conformations are observed to be 5 times more stable than the 4-membered complex. Protonation in turn stabilizes further the 4- and 6-membered complexes against their decomposition into reactants (*i.e.* BE for protonated 6-membered complex at NH_2 group is -37.2/-39.3 kcal $mole^{-1}$ while BE for non-protonated one is only -14.1/-14.6 kcal $mole^{-1}$, **Tables S1, S4**). Moreover, the 6-membered complexes with protonated NH_2 groups are even more stable than the final protonated product obtained during CO_2 protonation: [$PhCH_2NHCOOH$][$PhCH_2NH_3$]$^+$. Additionally, binding energies of neutral and protonated complexes between benzylamine and benzylcarbamic acid were

Table S6. Binding energies of neutral and protonated complexes between benzylamine and benzylcarbamic acid with respect to corresponding counterparts.

	$E_{tot} + E_{ZPE}$, a.u.	ω_1, cm^{-1}	BE, kcal mole^{-1}
**Counterpart A**<br><br>Benzylcarbamic acid anion<br>$[Ph\text{-}CH_2\text{-}NHCOO]^-$	-514.938213<br>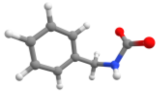	48	-
**Counterpart B**<br><br>Benzylamine cation<br>$[Ph\text{-}CH_2\text{-}NH_3]^+$	-327.217075<br>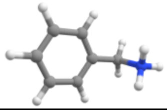	68	-
**Counterpart C**<br><br>Benzylcarbamic acid<br>$Ph\text{-}CH_2\text{-}NHCOOH$	-515.491933<br>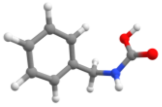	24	-
**Counterpart D**<br><br>Benzylamine<br>$Ph\text{-}CH_2\text{-}NH_2$	-326.860612<br>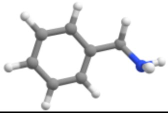	53	-
**Product 1.1**<br><br>Non-protonated *Conformation 1*<br>$[Ph\text{-}CH_2\text{-}NHCOOH][\ Ph\text{-}CH_2\text{-}NH_2]$	-842.368836<br>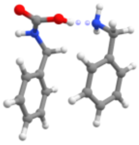	23	**A + B = Product 1.1**<br>-134 kcal mole^{-1}<br><br>**C + D = Product 1.1**<br>-10.2 kcal mole^{-1}
**Product 1.2**<br><br>Non-protonated *Conformation 2*<br>$[Ph\text{-}CH_2\text{-}NHCOOH][\ Ph\text{-}CH_2\text{-}NH_2]$	-842.374776<br>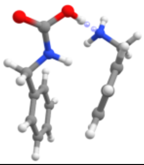	22	**A + B = Product 1.2**<br>-137 kcal mole^{-1}<br><br>**C + D = Product 1.2**<br>-14.0 kcal mole^{-1}
**Product 1.3**<br><br>Non-protonated *Conformation 3*<br>$[Ph\text{-}CH_2\text{-}NHCOOH][\ Ph\text{-}CH_2\text{-}NH_2]$	-842.378893<br>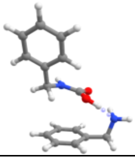	13	**A + B = Product 1.3**<br>-140 kcal mole^{-1}<br><br>**C + D = Product 1.3**<br>-16.5 kcal mole^{-1}
**Product 2.1**<br><br>Protonated *Conformation 1*<br>$[Ph\text{-}CH_2\text{-}NHCOOH][\ Ph\text{-}CH_2\text{-}NH_3]^+$	-842.759425<br>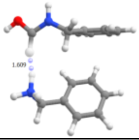	17	**C + B = Product 2.1**<br>-31.6 kcal mole^{-1}
**Product 2.2**<br><br>Protonated *Conformation 2*<br>$[Ph\text{-}CH_2\text{-}NHCOOH][\ Ph\text{-}CH_2\text{-}NH_3]^+$	-842.752104<br>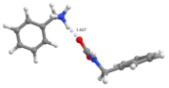	6	**C + B = Product 2.2**<br>-27.0 kcal mole^{-1}

compared with respect to their corresponding counterparts (**Table S6**). As can be seen, benzylcarbamic acid/benzylamine salt doesn't exist in a gas phase as ionic species. Instead, spontaneous intramolecular

proton transfer occurs during geometry optimization from NH_3^+ group to $NHCOO^-$ group to form complex between benzylcarbamic acid and benzylamine [Ph-CH_2-NHCOOH][Ph-CH_2-NH_2], which is stabilized by hydrogen bonding (BE of intramolecular protonation = 134−140 kcal $mole^{-1}$, **Table S6**). These findings suggest that protonation of NH_2 groups in the three-component complex as a six-membered species can be detected by the mass spectrometer. On the contrary, protonation of the intact benzylcarbamic acid/benzylamine salt was not feasible indicating its detection is not like. Instead, the individual components of this salt are detected independently in the mass spectrometer.

The stability of the protonated three-component complex in the form as a six-membered ring is seen by comparing the total energies (accounting of ZPE). For the protonated six-membered complex conformations, total energy, E_{tot} + E_{ZPE} = -842.771142 a.u. and -842.767929 a.u. (protonation at NH_2 groups) (**Table S4**). However, for two conformations of $[PhCH_2NHCOOH][PhCH_2NH_3]^+$, total energy E_{tot} + E_{ZPE} = -842.759425 a.u. and -842.752104 a.u. correspondingly (**Table S3**). Thus, the difference in E_{tot} + E_{ZPE} between the protonated product and protonated 6-membered complex is 0.008504–0.019038 a.u. (5.3–12 kcal $mole^{-1}$) in favor of higher stability for the protonated 6-membered complex.

It is noted that for conformation **(A)** of NH_2-protonated complex, we are not able to confirm the existence of 6-membered binding mode of CO_2 molecule, which is shifted predominantly to the $C_6H_5CH_2NH_3^+$ part of the complex (**Fig. 2A**, main manuscript). As a result, as it follows from Bader's QTAIM analysis, only one O atom of CO_2 is involved in the three non-covalent interactions with NH_3^+ group and aromatic C atoms of two benzylamine fragments (**Table S7**). Conformation **(B)** demonstrates a binding mode similar to the genuine 6-membered scheme, while QTAIM analysis does not indicate the existence on non-covalent O_2C---NH_2 interaction (**Fig. 2B**, main manuscript). Instead, each of Oxygen atoms coupled separately to the NH_2 and NH_3^+ groups forming something like 7-membered coupling. We speculate that, in some conformation, the C atom of CO_2 could form non-covalent interaction with Nitrogen atom of amine group similarly to non-protonated case in **Figure 1** (main manuscript). Moreover, if we consider the kinetics of chemical transformation of protonated complex towards products, the reaction coordinate itself assumes the formation of C-N covalent bond, so the presence or absence of non-covalent O_2C---NH_2 interaction is not mandatory for transformation of protonated complex into the product following a particular reaction coordinate.

Table S7. Bond lengths (*d*), energies (*E*), and the topological characteristics of the electron density distribution in the critical point (3, –1) for non-covalent interactions in NH_2-protonated collision complexes between benzylamine and CO_2.

Bond	d, Å	ρ(r), $e \cdot a_0^{-3}$	ν(r), *au*	g(r), *au*	h(r), *au*	$\nabla^2\rho(r)$, $e \cdot a_0^{-5}$	ε	DI	E, kcal $mole^{-1}$
**6-Membered Complex (Conformation *1*)**									
O1---H1	2.310	0.0122	-0.0081	0.0099	0.0018	0.04714	0.25	0.026	-2.54
N2---H2N1	1.733	0.0543	-0.0462	0.0343	-0.0119	0.0899	0.01	0.173	-14.50
O1---C1	3.325	0.0058	-0.0032	0.0039	0.0006	0.0180	1.50	0.020	-1.00
O1---C2	3.485	0.0045	-0.0021	0.0028	0.0007	0.0138	3.49	0.019	-0.66
**6-Membered Complex (Conformation *2*)**									
O1---N2	2.847	0.0100	-0.0073	0.0092	0.0019	0.0448	0.29	0.051	-2.29
O1---H4	3.133	0.0027	-0.0015	0.0020	0.0005	0.0101	0.38	0.010	-0.47
O2---N1	2.702	0.0060	-0.0039	0.0050	0.0011	0.0244	2.21	0.021	-1.22
O2---H3	2.783	0.0054	-0.0032	0.0040	0.0008	0.0192	0.23	0.019	-1.00
N2H2---N1	1.762	0.0508	-0.0420	0.0322	-0.0098	0.0896	0.01	0.164	-13.18

10. Determination of CO_2 Capture Capacity of Different Amines by Contained-SESI

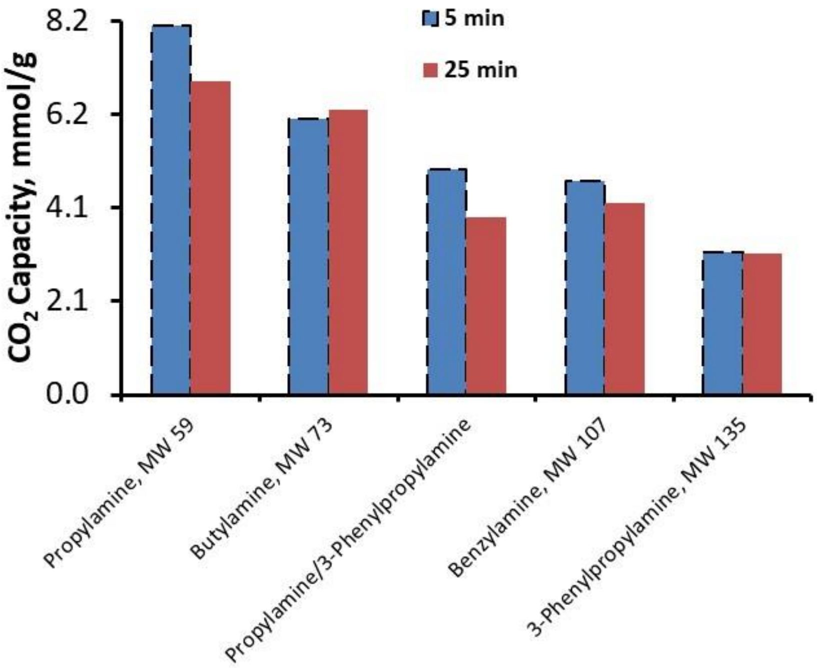

Figure S11. Bar graph displaying the CO_2 capture capacity in mmol/g of different amines for two reaction times using contained-SESI. It is shown that 5 min reaction time yields better capacities for almost all amines compared to 25 min.

11. Influence of Molecular Weights of Amines on their CO_2 Capture Capacity by Contained-SESI

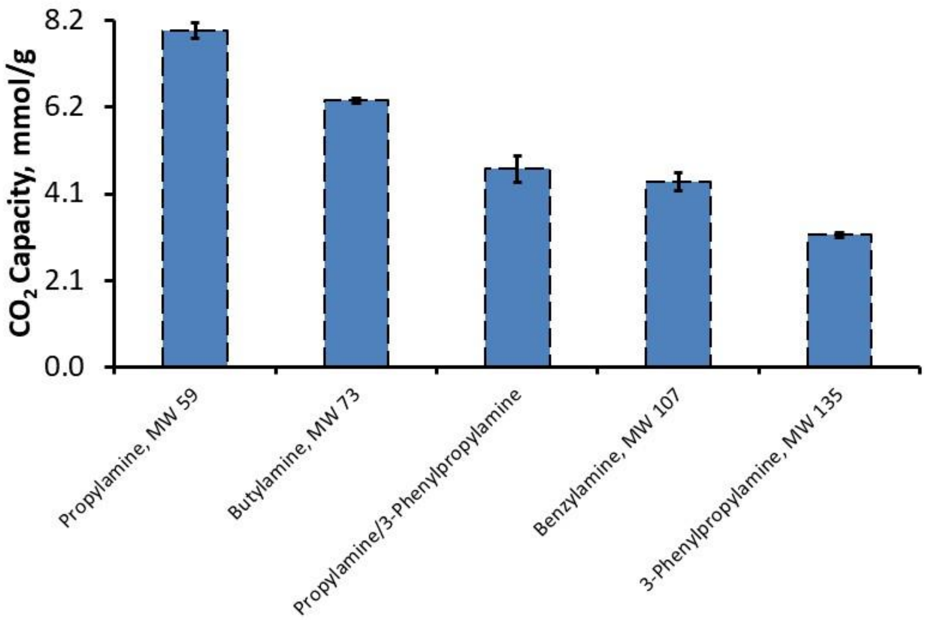

Figure S12. Bar graph showing the CO_2 capture capacity in mmol/g for different amines using contained-SESI. As can be seen the amines with smaller molecular weights have higher CO_2 capture capacities compared to amines with higher molecular weights.

12. Cavity Size Effect on the Product Formation

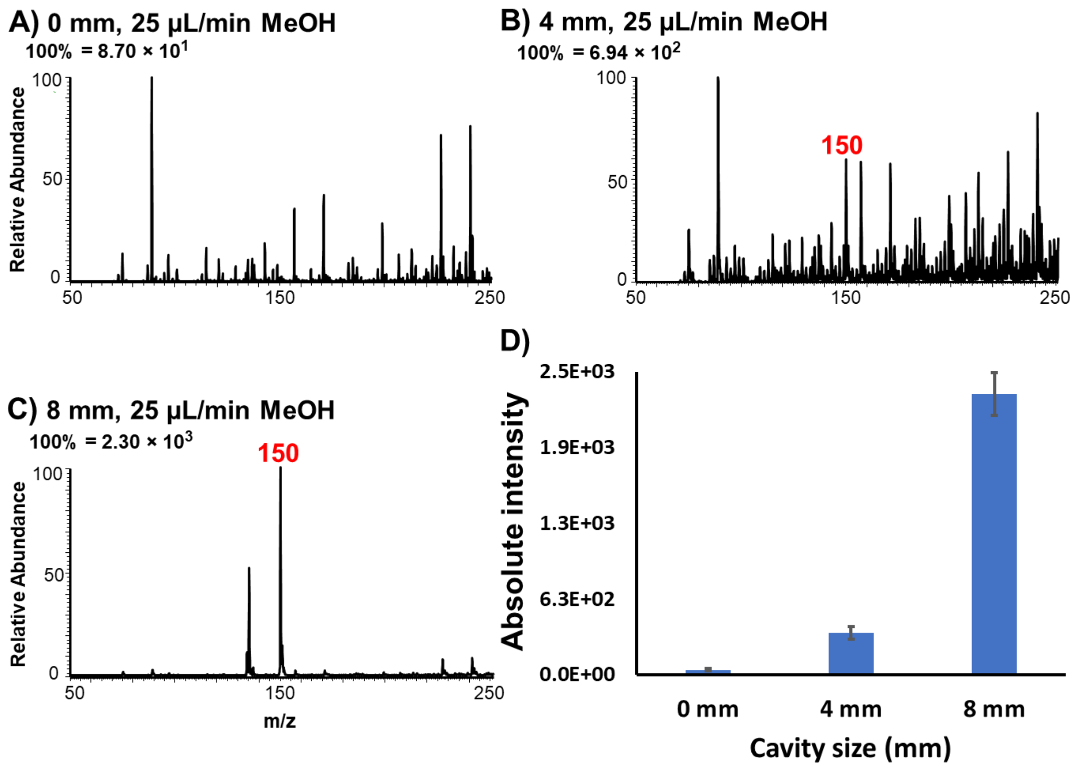

Figure S13. The effect of cavity size on the product formation of reaction between benzylamine and CO_2; **A)** 0 mm **B)** 4 mm, and **C)** 8 mm cavity sizes. **D)** the bar graph showing the product absolute intensity *vs* cavity size in mm.

13. Solvent and Temperature Effect on the Benzylamine Vapor Fragmentation by Contained-SESI

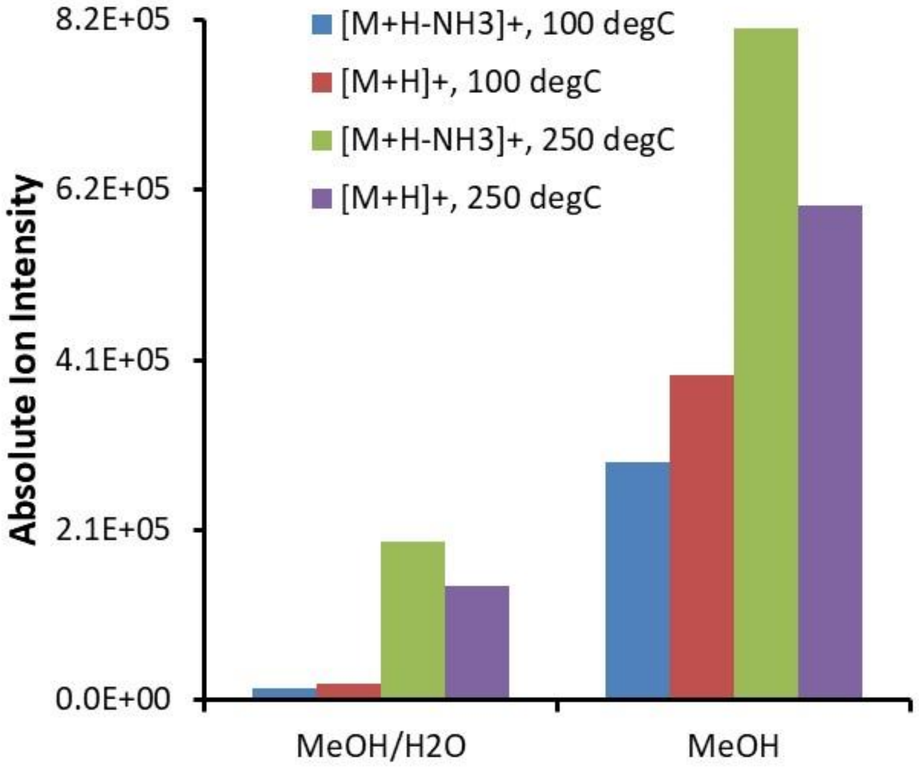

Figure S14. Bar graph showing the effect of solvent and temperature on the benzylamine vapor fragmentation by contained-SESI. In full mass spectrum, the protonated benzylamine at *m/z* 108, $[M + H]^+$, undergoes a neutral loss of ammonia (NH_3) to afford the peak at m/z 91 which is solvent and temperature dependent.

14. Optimizations of Solvent Flowrate, Gradient Flowrate, and Spray Voltage for Butylcarbamate Product Formation in Contained-SESI

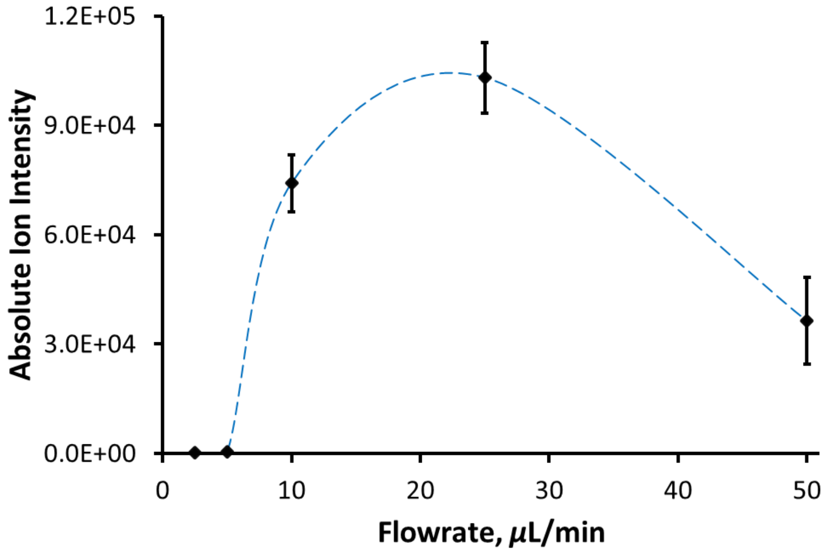

Figure S15. Graph demonstrating the effect of the MeOH solvent flowrate on the formation of butylcarbamate product, *m/z* 116 $[M - H + CO_2]^-$, formation showing an optimized flowrate of 25 μL/min. Other parameters include: 40 psi N_2 nebulizer gas, 250 °C MS inlet capillary temperature, and 5 kV spray voltage.

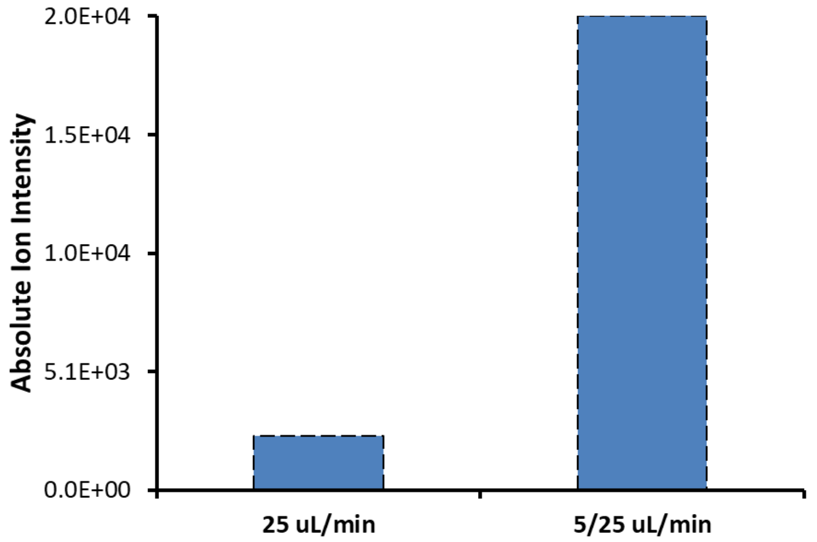

Figure S16. Bar graph showing the effect of constant flowrate *vs*. gradient flowrate. The results show that adopting a gradient flowrate by switching between 5 and 25 μL/min generates the higher absolute intensity for the product, benzylcarbamate.

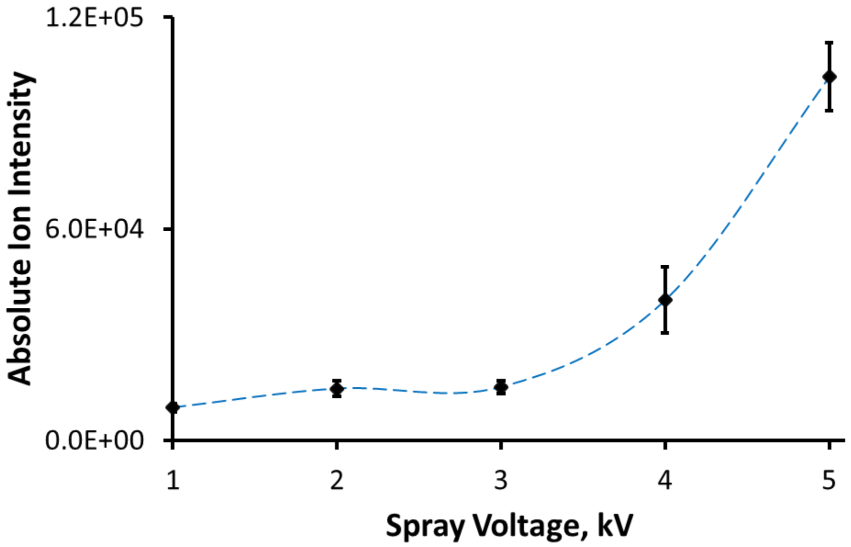

Figure S17. Graph showing the effect of spray voltage on contained-SESI formation of butylcarbamate product, *m/z* 116 $[M\text{-}H + CO_2]^-$. The results show that 5 kV generates the highest absolute intensity. Other parameters include: 25 µL/min MeOH spray solvent flowrate, 40 psi N_2 nebulizer gas, and 250 °C MS inlet capillary temperature.

15. Spray Voltage Effect in Contained-SESI

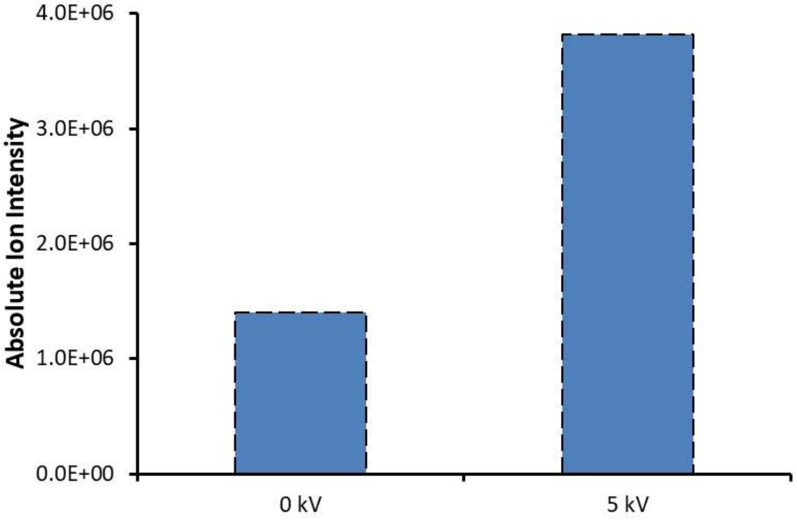

Figure S18. Bar graph showing the effect of spray voltage on benzylamine vapor detection in positive ion mode and with no cavity of contained-secondary ESI. The Y axis shows the sum of absolute intensities of protonated amine peak at *m/z* 108 $[M + H]^+$ and its fragment ion after loss of ammonia in full mass spectrum at *m/z* 91 $[M + H - NH_3]^+$. Other parameters include: 25 µL/min MeOH spray solvent flowrate, 150 psi N_2 nebulizer gas, and 250 °C MS inlet capillary temperature.

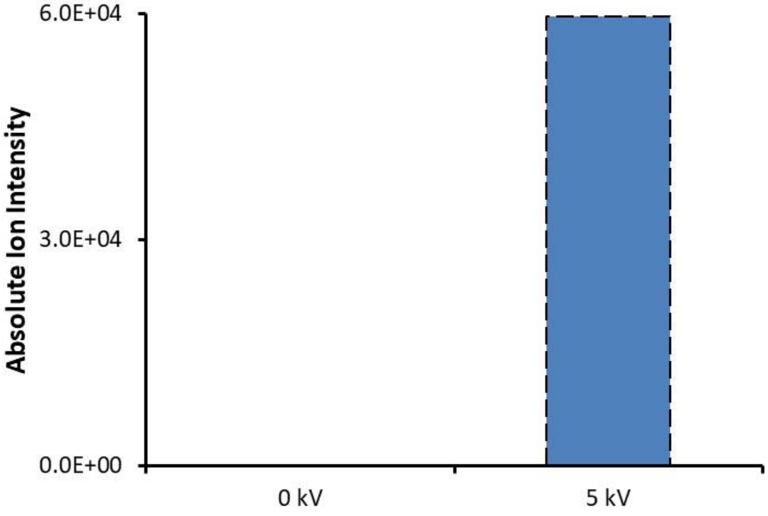

Figure S19. Bar graph showing the effect of spray voltage on benzylcarbamate product formation, *m/z* 150 $[M – H + CO_2]^–$, from the reaction between benzylamine and CO_2 in negative ion mode and with 8 mm cavity size in contained-secondary ESI. Other parameters include: 5/25 µL/min gradient for MeOH spray solvent flowrate, 40 psi N_2 nebulizer gas, and 250 °C MS inlet capillary temperature.

16. N_2 Gas Pressure Optimization for Butylcarbamate Product Formation in Contained-SESI

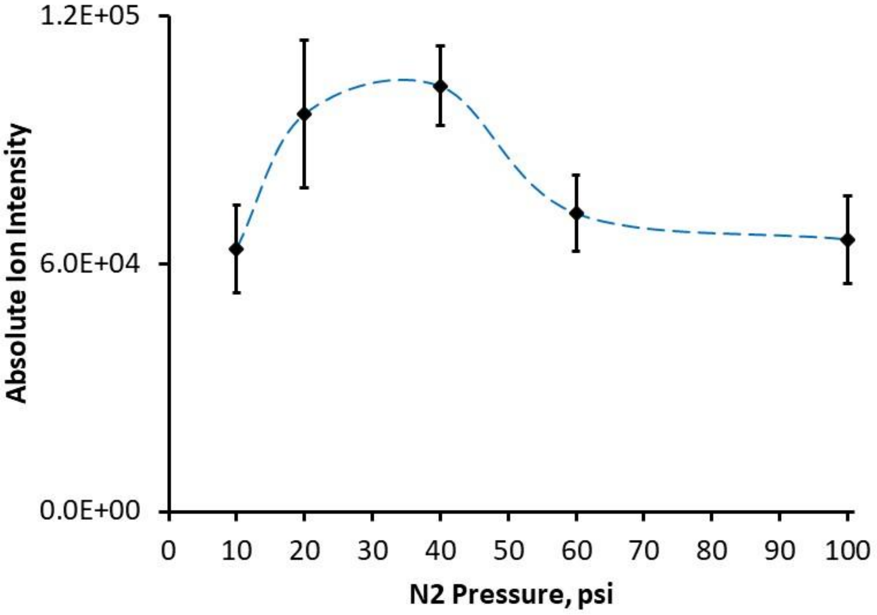

Figure S20. Graph demonstrating the optimization of N_2 nebulizer gas for the butylcarbamate product formation, *m/z* 116 $[M - H + CO_2]^-$, from the reaction between butylamine and CO_2 in negative ion mode and with 8 mm cavity size in contained-secondary ESI. It was determined that the 40 psi N_2 nebulizer gas pressure yielded the highest product intensity. Other parameters include: 25 μL/min MeOH spray solvent flowrate, 250 °C MS inlet capillary temperature, and 5 kV spray voltage.

17. N_2 or CO_2 Nebulizer Gas Effects on Benzylcarbamate Product Formation in Contained-SESI

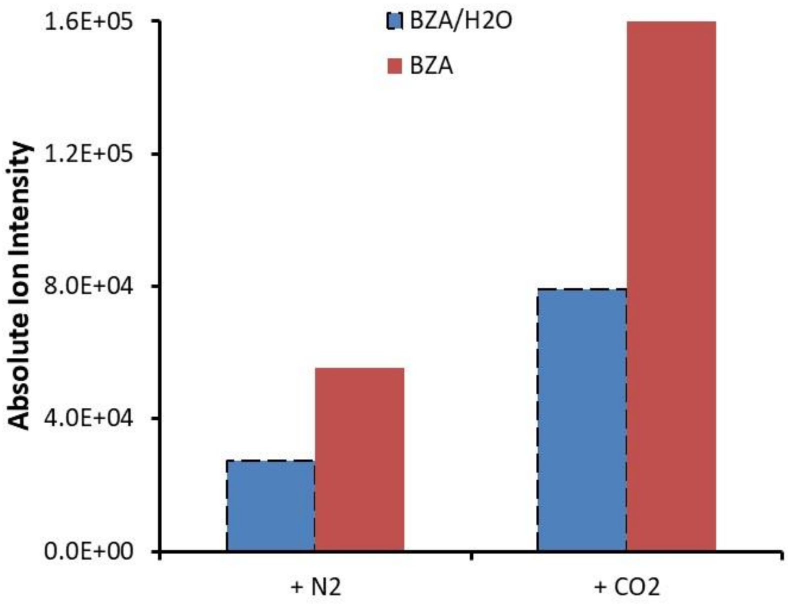

Figure S21. Bar graph demonstrating the effect of N_2 or CO_2 nebulizer gases for the benzylcarbamate product formation, *m/z* 150 $[M - H + CO_2]^-$, from the reaction between benzylamine and CO_2 in negative ion mode and with 8 mm cavity size in contained-SESI. It is shown that pure amine and CO_2 as the nebulizer gas led to the highest amount of product, however, the solution of amine/H_2O (1:1 v/v) can also result in the formation of product. Other parameters include: 5/25 µL/min gradient for MeOH spray solvent flowrate, 40 psi nebulizer gas pressure, 250 °C MS inlet capillary temperature, and 5 kV spray voltage.

18. Comparison of Different Ionization Techniques on Benzylcarbamate Formation

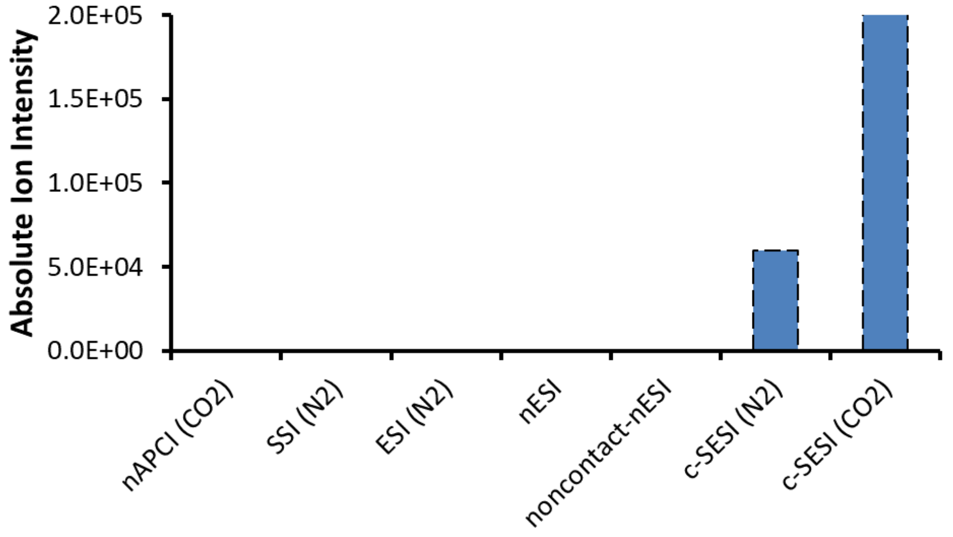

Figure S22. Comparison of different ionization techniques on the formation of benzylcarbamate product, *m/z* 150 [M – H + CO_2]$^-$, from the reaction between benzylamine (20 µM) and CO_2 in negative ion mode.

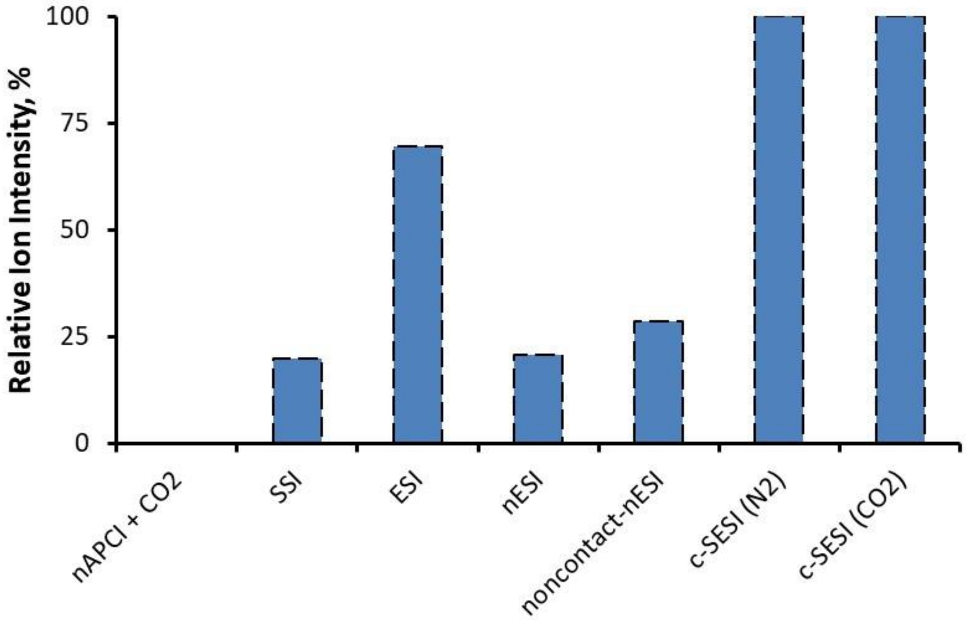

Figure S23. Comparison of different ionization techniques on the formation of benzylcarbamate product, *m/z* 150 [M – H + CO_2]$^-$, from the reaction between benzylamine (10 mM) and CO_2 in negative ion mode.

19. Optimization of *Online* CO_2 Capture

The initial sets of experiments involved *online* monitoring of CO_2 capture products by mass spectrometry, as controlled by different modes of operating the contained-SESI source. In this way, we were able to optimize the performance of the platform with ease, while also offering a quantitative way to measure the amount of amine vapor that engages in the CO_2 capture/reaction. The *online* monitoring/optimization is discussed first, followed by vapor quantification. The cavity size included at the outlet of the contained-SESI source was optimized by adjusting the inner capillary, which ranged from Type I (no cavity) to Type II operational modes. The negative-ion mode mass spectra signals derived from the exposure of benzylamine vapor and CO_2 gas are shown in **Figure S12**, with emphasis placed on carbamic acid (MW 151 Da) reaction product detected at *m/z* 150.

The results show that in the absence of a cavity (**Figure S13A**) formation of the carbamic acid product through the capture of CO_2 by the amine is not favored. However, obvious signal for the product is detected upon the creation of the reaction cavity (4 mm), which was significantly increased by further increase of the cavity length to 8 mm. Signal decreased was observed for cavity length longer than 8 mm (**Figure S13B** and **C**). Hence, the 8 mm cavity size was selected for subsequent experiments.

Our previous investigations[12,13] indicated the presence of both (bigger) droplets and thin liquid films inside the cavity, which further breaks up to yield secondary microdroplets upon reaching the tip of the outer capillary. Thus, we have two distinct micro-reactors (droplets and liquid thin film) capable of accelerating chemical reactions due to confinement and concentration effects. Mixing of amine vapor and CO_2 gas into the liquid thin film/droplets occurs more effectively in the contained-SESI setup because of the turbulent flow effect induced by inserting the inner electrospray emitter into the cavity.[14] Additionally, the introduction of high-pressure gas into the cavity assists droplet/thin film desolvation, reducing droplet speed (due to cavity), which subsequently facilitate reaction by increasing reagent residence time in the droplet/thin film reaction environment. The confined space of the cavity causes more effective collisions between reacting species, compared to traditional ESI plume sprayed into ambient air where some reagents can easily dissipate. All these interesting features (*i.e.*, confinement, concentration, evaporation, and turbulent mixing) make the presence of cavity in the contained-SESI source a superior reaction system when compared with other reaction systems that utilize droplet/gas interactions (*e.g.*, those based on either charged droplets or thin films alone).

Aside from cavity length, the spray solvent composition and inlet capillary temperate were also optimized, in which we tested the reactivity and capture capabilities of droplets/thin films derived from pure MeOH and MeOH/H_2O at two different inlet temperatures. This solvent choice was based on the fact that MeOH and H_2O are commonly used protic solvents in secondary ESI enabling effective protonation/deprotonation of gas-phase analytes. **Figures S3 and S13** show that pure MeOH and 250 °C were the best spray solvent and MS inlet capillary temperature, respectively. From a mechanistic standpoint, a higher temperature can increase the evaporation and desolvation of charged droplets, thus enhancing the delivery of ions into the mass spectrometer for detection. The spray solvent flowrate was also optimized, and it turned out that 25 μL/min produced the highest product signal as shown in **Figures S14**. The optimized flowrate (25 μL/min) was then tested in a gradient fashion by periodic switching of flowrate between two values of 5 and 25 μL/min (6 sec for each flowrate). Results from this flow switching experiment indicated that the gradient flowrate provides a significant increase in the product signal intensity (1−2 orders of magnitude) than just utilizing a constant flowrate (**Figure S15**). Such gradient effect presumably could be explained by regeneration of oversaturated thin films inside of the reaction cavity, in

terms of fresh solvent and new portion of reaction products. Also, optimization of spray voltage revealed that ±5 kV resulted in the highest signal intensity for both amine and its CO_2 product (**Figures S16−S18**). It is noteworthy that N_2 can be used instead of CO_2 as nebulizing gas and still observe product formation. In this case, the N_2 pressure was optimized to be 40 psi (**Figure S19**). When using N_2 as nebulizing gas, the absolute product $[M - H + CO_2]^-$ ion intensity at *m/z* 150 derived from headspace vapors of both pure benzylamine and its corresponding aqueous solutions differed by only 3X in favor of pure amine (**Figure S20**). In other words, headspace vapor of amine solutions (not the neat amine), with limited volatility, can be used in the contained-SESI experiment for CO_2 capture studies (analysis of less volatile amines is discussed later). Interestingly, we observed similar 3X signal improvement for experiments that utilized CO_2 as nebulizer gas (**Figure S20**), for neat amine *versus* amine solution.

To estimate the concentration of amine vapor introduced to mass spectrometer, we performed an external calibration experiment in which several solutions of benzylamine were prepared at different concentrations (1-100 μM). We then analyze the solutions using ESI (the same contained-SESI was used but in Type III mode), which revealed that the concentration of amine vapor captured and introduced to the mass spectrometer in our typical contained-SESI experiment (Type II mode with 8 mm cavity) was approximately 20 μM. To compare the reactivity of our method to other gas-phase and droplet-based ionization techniques, the 20 μM solution of benzylamine was analyzed by atmospheric pressure chemical ionization (**APCI**; with different discharge gases: Air, CO_2, and N_2), sonic spray ionization (**SSI**; no spray voltage, but N_2 nebulizer gas was used), **ESI** (with N_2 nebulizer gas), **nano-ESI**, and **noncontact nano-ESI**. All these methods failed to enable benzylamine/CO_2 reaction. Under similar experimental conditions (20 μM benzylamine solution), however, our optimized contained-SESI platform (Type II mode with 8 mm reaction cavity) afforded a product detected *m/z* 150 at 10^4 signal level (with N_2 nebulizer gas) and 10^5 level (with CO_2 nebulizer gas), as summarized in **Figure S21**. Remarkably, increasing benzylamine concentration to 10 mM (as used by other CO_2 capture experiments)[13,14] for the other methods mentioned above (APCI, SSI, ESI, nano-ESI, noncontact nano-ESI) did not provide a dominant capture product at *m/z* 150 in the resultant mass spectra. On the contrary, the $[M - H + CO_2]^-$ product was predominant species detected in our contained-SESI approach (**Figure S22**), either using CO_2 or N_2 as the nebulizer gas. These results testify to the high sensitivity and reactivity of our contained-SESI method, which can be ascribed to the softness, enabling the preservation of the integrity of labile compounds as well as the less matrix effect associated with the use of headspace vapor of amines *versus* directly electrospraying amines dissolved into bulk solution-phase.

20. Full Mass Spectrum of Benzylcarbamate from High-throughput Analysis by Contained-SESI

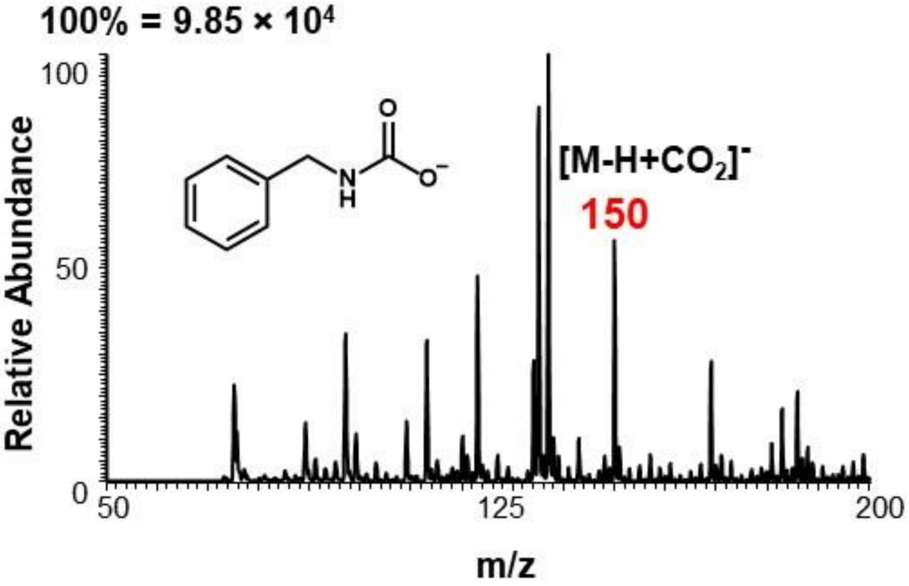

Figure S24. Negative mode full mass spectrum of the benzylcarbamate product from the high-throughput analysis of the reaction between benzylamine headspace vapor and CO_2 by contained-SESI with 8 mm cavity size.

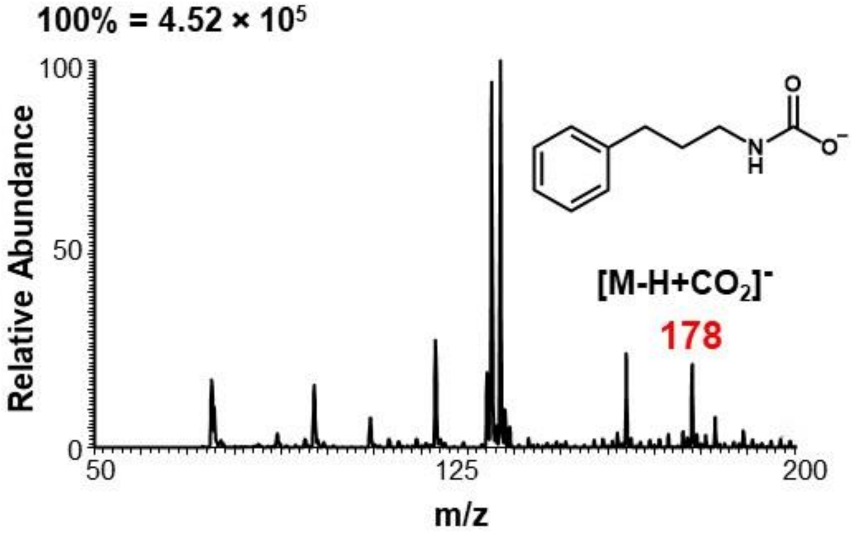

Figure S25. Negative mode full mass spectrum of the 3-phenylpropylcarbamate product from the high-throughput analysis of the reaction between 3-phenylpropylamine headspace vapor and CO_2 by contained-SESI with 8 mm cavity size.

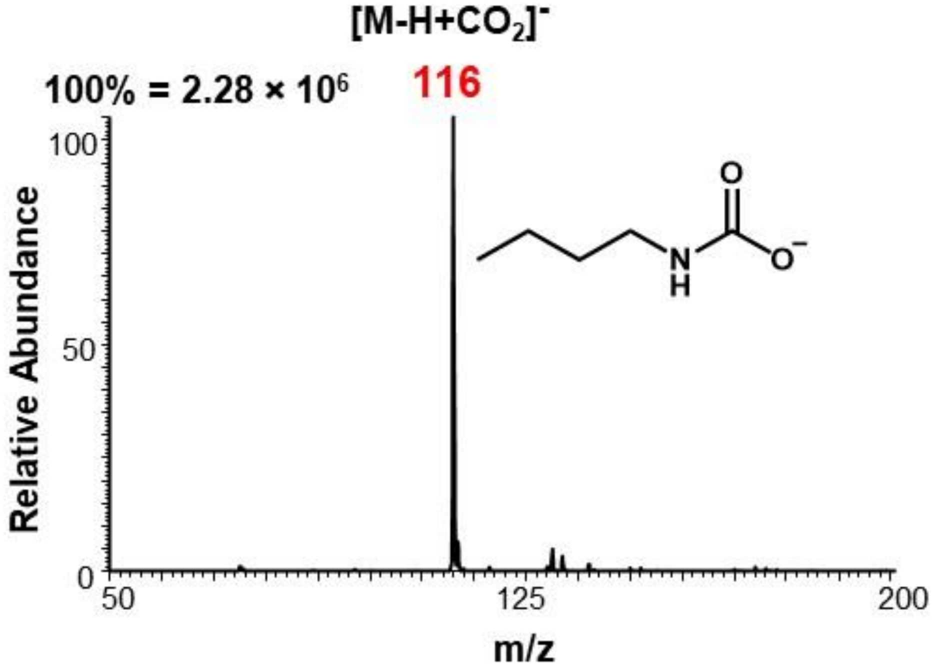

Figure S26. Negative mode full mass spectrum of the butylcarbamate product from the high-throughput analysis of the reaction between butylamine headspace vapor and CO_2 by contained-SESI with 8 mm cavity size.

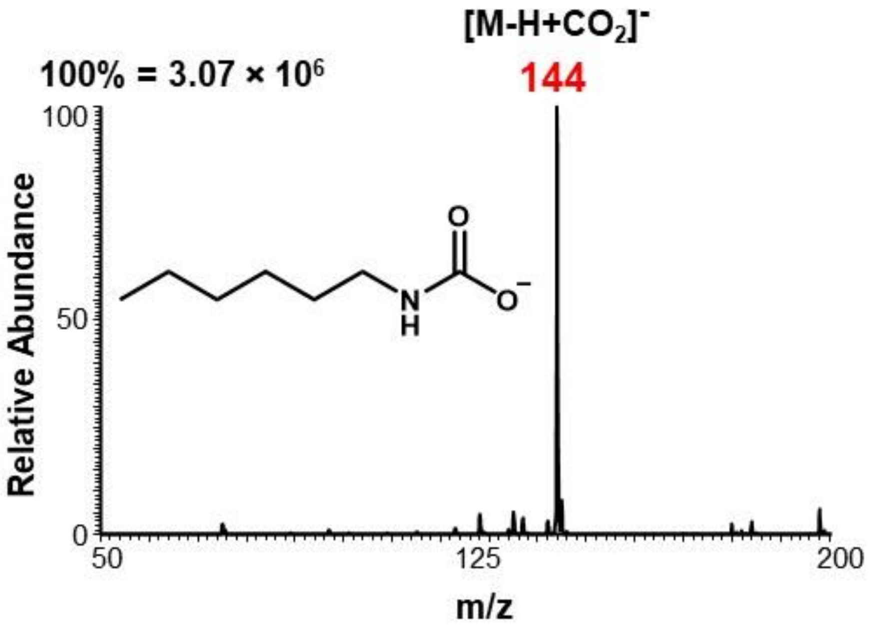

Figure S27. Negative mode full mass spectrum of the hexylcarbamate product from the high-throughput analysis of the reaction between hexylamine headspace vapor and CO_2 by contained-SESI with 8 mm cavity size.

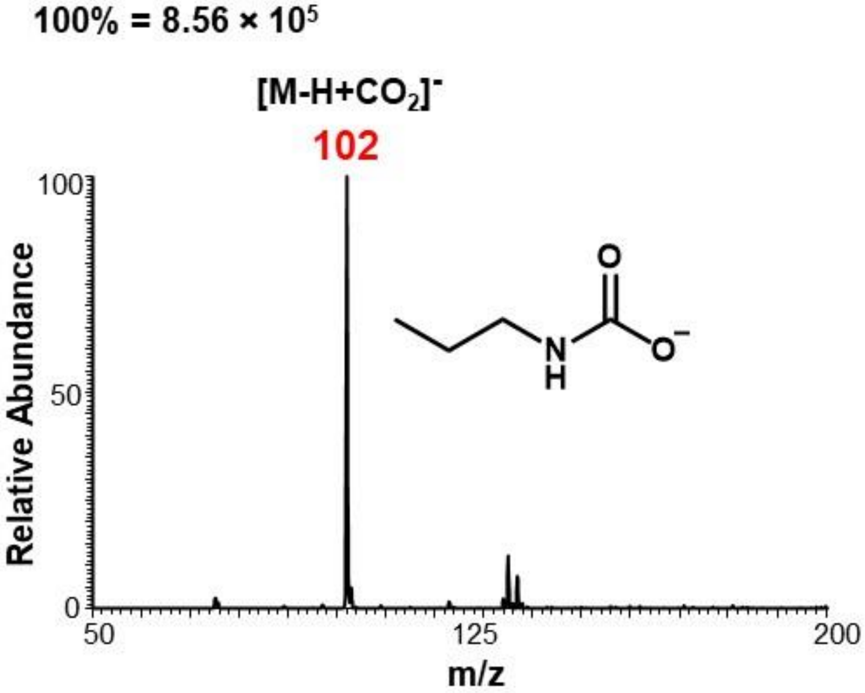

Figure S28. Negative mode full mass spectrum of the propylcarbamate product from the high-throughput analysis of the reaction between propylamine headspace vapor and CO_2 by contained-SESI with 8 mm cavity size.

21. Effect of Solvent Addition on the Formation of 3-Phenylpropylcarbamate by Contained-SESI

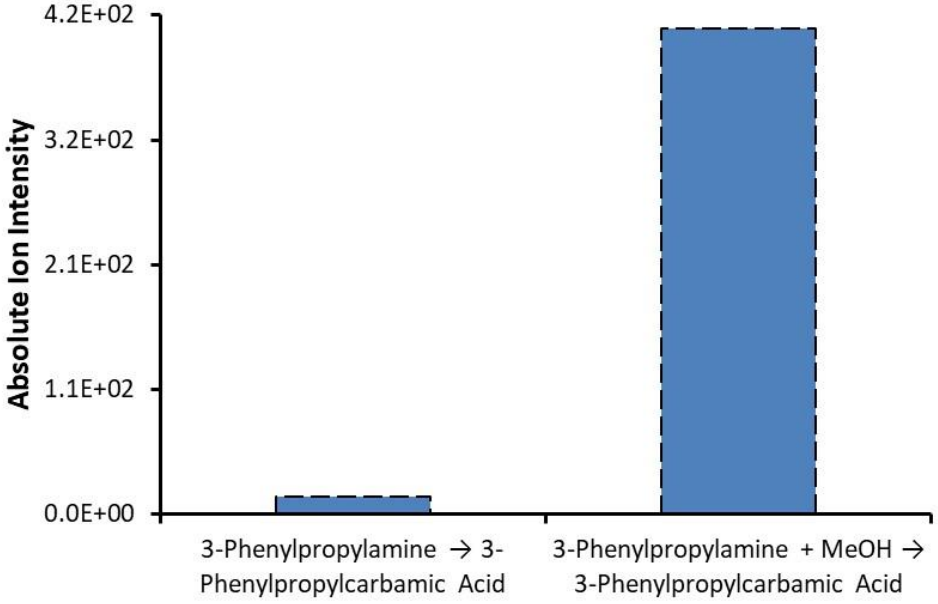

Figure S29. Solvent effect on the signal intensity of the 3-phenylpropylcarbamate product, *m/z* 178 $[M - H + CO_2]^-$ from the reaction between headspace vapor arising from the solution of 3-phenylpropylamine mixed with MeOH (10:1 v/v) and CO_2 by contained-SESI with 12 mm cavity size. Addition of small amount of MeOH increases the vapor pressure of the amine and hence the product formation. Other parameters include: 5/25 µL/min gradient for MeOH spray solvent flowrate, 40 psi N_2 nebulizer gas pressure, 250 °C MS inlet capillary temperature, and 5 kV spray voltage.

22. Solvent Addition Effect on the Vapor Pressure of Dodecylamine by Contained-SESI

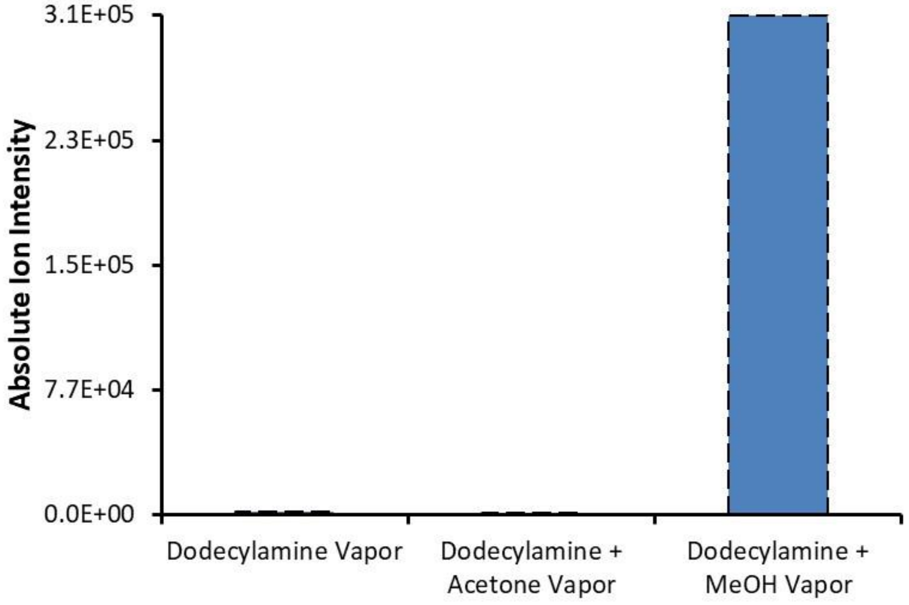

Figure S30. Solvent effect on the signal intensity of protonated dodecylamine, *m/z* 186 $[M + H]^+$ from the headspace vapor arising from the solution of pure dodecylamine or dodecylamine mixed with MeOH, or acetone (10:1 v/v) and CO_2 by contained-SESI with 8 mm cavity size. Addition of small amount of MeOH increases the vapor pressure of the amine. However, the same effect was not observed for acetone solvent. Other parameters include: 5/25 µL/min gradient for MeOH spray solvent flowrate, 40 psi N_2 nebulizer gas pressure, 250 °C MS inlet capillary temperature, and 5 kV spray voltage.

23. Effect of Cavity Size on Diamine Carbamate Product Formation by Contained-SESI

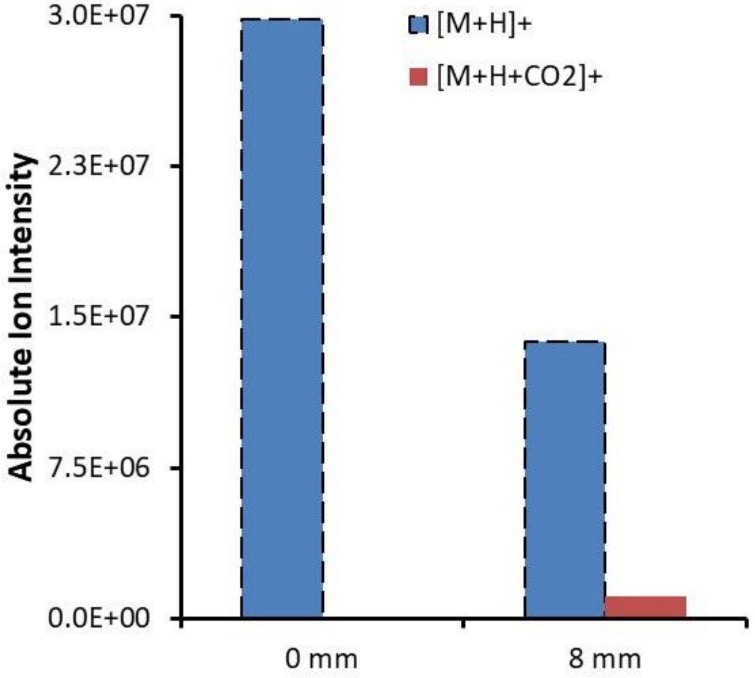

Figure S31. The cavity size effect on the signal intensities of 10 mM N,N-dibutyl-1,3-propanediamine (DBPA), *m/z* 186 $[M + H]^+$, in ACN/MeOH or formation of DBPA carbamate product, *m/z* 231 $[M + H + CO_2]^+$ from the reaction of DBPA headspace vapor and CO_2. Other parameters include: 5 µL/min ACN/MeOH spray solvent flowrate, 40 psi N_2 nebulizer gas pressure, 250 °C MS inlet capillary temperature, and 5 kV spray voltage.

24. References

(1) Becke, A. D. Density-functional Thermochemistry. III. The Role of Exact Exchange. *The Journal of Chemical Physics* **1993**, *98* (7), 5648–5652. https://doi.org/10.1063/1.464913.

(2) Lee, C.; Yang, W.; Parr, R. G. Development of the Colle-Salvetti Correlation-Energy Formula into a Functional of the Electron Density. *Phys. Rev. B* **1988**, *37* (2), 785–789. https://doi.org/10.1103/PhysRevB.37.785.

(3) McLean, A. D.; Chandler, G. S. Contracted Gaussian Basis Sets for Molecular Calculations. I. Second Row Atoms, Z=11–18. *The Journal of Chemical Physics* **2008**, *72* (10), 5639–5648. https://doi.org/10.1063/1.438980.

(4) Frisch, M. J.; Pople, J. A.; Binkley, J. S. Self-consistent Molecular Orbital Methods 25. Supplementary Functions for Gaussian Basis Sets. *The Journal of Chemical Physics* **1984**, *80* (7), 3265–3269. https://doi.org/10.1063/1.447079.

(5) Grimme, S.; Antony, J.; Ehrlich, S.; Krieg, H. A Consistent and Accurate Ab Initio Parametrization of Density Functional Dispersion Correction (DFT-D) for the 94 Elements H-Pu. *J Chem Phys* **2010**, *132* (15), 154104. https://doi.org/10.1063/1.3382344.

(6) *Gaussian 16 Rev. B.01 Release Notes | Gaussian.com*. https://gaussian.com/relnotes_b01/ (accessed 2023-08-16).

(7) Bader, R. F. W. *Atoms in Molecules: A Quantum Theory*; International Series of Monographs on Chemistry; Oxford University Press: Oxford, New York, 1994.

(8) Bader, R. F. W. Atoms in Molecules. *Acc. Chem. Res.* **1985**, *18* (1), 9–15. https://doi.org/10.1021/ar00109a003.

(9) Bader, R. F. W. A Quantum Theory of Molecular Structure and Its Applications. *Chem. Rev.* **1991**, *91* (5), 893–928. https://doi.org/10.1021/cr00005a013.

(10) *Some References Related to AIMAll*. https://aim.tkgristmill.com/references.html (accessed 2024-10-02).

(11) Espinosa, E.; Molins, E.; Lecomte, C. Hydrogen Bond Strengths Revealed by Topological Analyses of Experimentally Observed Electron Densities. *Chemical Physics Letters* **1998**, *285* (3), 170–173. https://doi.org/10.1016/S0009-2614(98)00036-0.

(12) Miller, C. F.; Kulyk, D. S.; Kim, J. W.; Badu-Tawiah, A. K. Re-Configurable, Multi-Mode Contained-Electrospray Ionization for Protein Folding and Unfolding on the Millisecond Time Scale. *Analyst* **2017**, *142* (12), 2152–2160. https://doi.org/10.1039/C7AN00362E.

(13) Miller, C. F.; Burris, B. J.; Badu-Tawiah, A. K. Spray Mechanism of Contained-Electrospray Ionization. *J. Am. Soc. Mass Spectrom.* **2020**, *31* (7), 1499–1508. https://doi.org/10.1021/jasms.0c00044.

(14) Badu-Tawiah, A. K.; Campbell, D. I.; Cooks, R. G. Accelerated C–N Bond Formation in Dropcast Thin Films on Ambient Surfaces. *J. Am. Soc. Mass Spectrom.* **2012**, *23* (9), 1461–1468. https://doi.org/10.1007/s13361-012-0394-y.
